# Systems-level restoration of vaginal and gut microbiota by *Lactobacillus helveticus* 20838 alleviates *Gardnerella vaginalis*-induced dysbiosis

**DOI:** 10.1093/ismejo/wrag118

**Published:** 2026-05-17

**Authors:** Juewon Kim, Woo-il Kim, Kyuyeon Lee, Nayeon Kim, Ah Young Hwang, Dong Ho Suh, Donghyun Cho, Yosep Ji, Hye-Ji Kang, Eun Sung Jung

**Affiliations:** Department of Physiology, Konkuk University College of Medicine, Chungju 27478, Republic of Korea; Department of Physiology, Konkuk University College of Medicine, Chungju 27478, Republic of Korea; HEM Pharma Inc., Suwon 16506, Republic of Korea; HEM Pharma Inc., Suwon 16506, Republic of Korea; HEM Pharma Inc., Suwon 16506, Republic of Korea; HEM Pharma Inc., Suwon 16506, Republic of Korea; HEM Pharma Inc., Suwon 16506, Republic of Korea; HEM Pharma Inc., Suwon 16506, Republic of Korea; HEM Pharma Inc., Suwon 16506, Republic of Korea; Microbiome Convergence Research Center, Korea Research Institute of Bioscience and Biotechnology (KRIBB), Daejeon 34141, Republic of Korea; HEM Pharma Inc., Suwon 16506, Republic of Korea

**Keywords:** vaginal dysbiosis, gut-vagina axis, *Lactobacillus helveticus*, vaginal microbiome, microbiota restoration

## Abstract

Bacterial vaginosis (BV), often driven by *Gardnerella vaginalis* overgrowth, is characterized by epithelial disruption, inflammation, and microbiome dysbiosis across the vaginal and gut ecosystems. Affecting a majority of women during their reproductive years, BV increases the risk of infection and reproductive complications. Here, we identify a novel probiotic strain, *Lactobacillus helveticus* 20838, exhibiting potent antagonistic activity against *G. vaginalis* and evaluate its ecological and immunological effects in a murine model of vaginitis. Comparative genomics revealed distinct adaptive and antimicrobial traits of *L. helveticus* 20838 relatives to the reference strain DPC4571. Both oral and intravaginal administration reduced *G. vaginalis* colonization, suppressed *Tnf-α* and *Il-1β* expression in vaginal tissue, and prevented pathological epithelial thickening. Multi-omics profiling of fecal and vaginal samples demonstrated restoration of microbial alpha and beta diversity disrupted by infection. The *L. helveticus* 20838 reduced dysbiosis-associated taxa such as *Staphylococcaceae*, whereas enriching protective *Lactobacillus* species, with intravaginal delivery achieving superior local recolonization of *Lactobacillaceae*. Collectively, these findings identify *L. helveticus* 20838 as a next-generation probiotic that alleviates *G. vaginalis*–induced dysbiosis by restoring microbial and immune homeostasis across interconnected mucosal niches, providing a systems-level framework for microbiota-targeted therapy in women’s health.

## Graphical Abstract

**Graphical Abstract:**
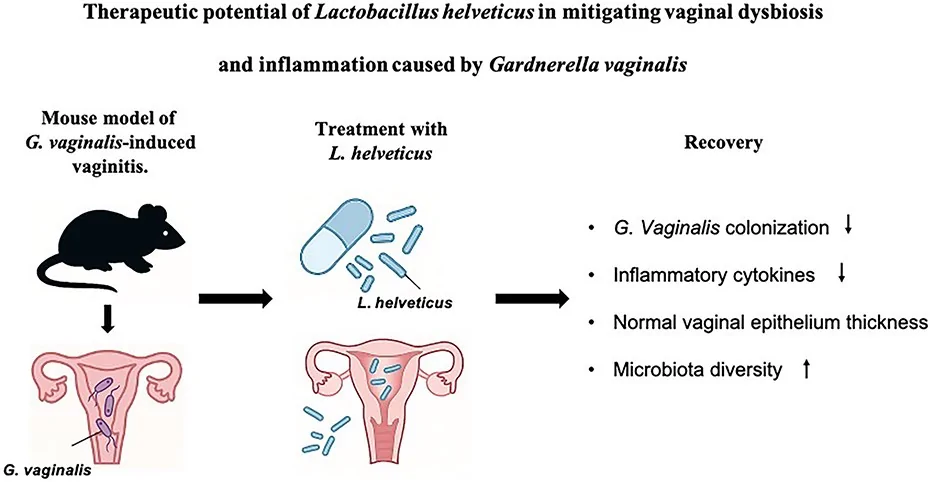

## Introduction

Vaginitis, a multifactorial condition presenting with irritation, discharge, malodor, and discomfort, is among the most common reasons for gynecologic consultation worldwide [1, 2]. Its etiology includes both infectious and non-infectious causes, such as microbial pathogens, hormonal changes, chemical irritants, and trauma [3, 4]. Vaginitis refers to inflammation of the vagina, which may arise from infectious agents such as bacterial vaginosis (BV), trichomoniasis, and vulvovaginal candidiasis, or from non-infectious factors including atrophic changes, irritants, or allergic responses. Infectious vaginitis is typically caused by microbial pathogens and represents the most common clinical category, whereas non-infectious vaginitis encompasses conditions such as atrophic and irritation-induced inflammation. Accurate clinical characterization requires integration of symptoms, exam findings, and diagnostic testing [5–10]. Because clinical manifestations overlap across subtypes, accurate diagnosis requires both clinical and laboratory evaluation [11]. BV is particularly prevalent, affecting up to 70% of women during their reproductive lifespan. It is defined by the depletion of protective *Lactobacillus* species, overgrowth of anaerobic pathogens, local inflammation, and heightened risk of adverse reproductive outcomes, including sexually transmitted infections, pelvic inflammatory disease, and preterm birth [9].

The vaginal microbiota plays a central role in protecting reproductive health. In healthy women, *Lactobacillus* species dominate, producing lactic acid to maintain an acidic vaginal environment, thereby suppressing pathogen colonization and reducing risks of BV and sexually transmitted infections [12, 13]. Disruption of this ecosystem, termed vaginal dysbiosis, is strongly associated with *G. vaginalis* overgrowth, a biofilm-forming bacterium that resists antibiotics and host defenses and produces virulence factors such as vaginolysin, which compromises epithelial integrity and provokes inflammation [14–16]. Standard antibiotic therapies, including metronidazole and clindamycin, are effective initially but often fail to prevent recurrence and may further disturb beneficial microbial communities [17]. These limitations underscore the urgent need for microbiota-preserving alternatives.

Probiotics have emerged as promising candidates for microbiome-targeted interventions. Clinical and preclinical studies demonstrate that selected *Lactobacillus* strains can alleviate BV symptoms, reinforce epithelial integrity, and downregulate pro-inflammatory responses [18, 19]. Among them, *L. helveticus* has shown potential to improve BV-associated outcomes in vivo, including reduction of *G. vaginalis* burden and inflammatory markers in murine models [20]. However, its therapeutic efficacy against *G. vaginalis*-induced vaginitis remains unexplored. In this study, we characterized a novel strain, *L. helveticus* 20838 (KCCM13578P), which was first isolated from Greek yogurt collected in Pohang, Republic of Korea. We then evaluated its probiotic potential in a murine model of *G. vaginalis*-induced vaginitis. Using both oral and intravaginal administration, we assessed its capacity to suppress pathogen burden, modulate inflammatory gene expression, preserve vaginal epithelial structure, and restore microbial community dynamics through histological and 16S rRNA gene-based analyses. Our findings provide new insights into *L. helveticus* 20838 as a microbiome-targeted therapeutic for restoring vaginal health.

## Materials and methods

### Study approval and ethical compliance

All animal procedures were conducted in accordance with institutional guidelines and approved by the Institutional Animal Care and Use Committee of HLB BioStep (IACUC No. BIOSTEP IACUC 23-KE-0509). Animals were maintained under specific pathogen-free (SPF) conditions, and all efforts were made to minimize animal suffering.

### Experimental model and study participant details

Six-week-old female SPF C57BL/6 mice were purchased from Koatech (Pyeongtaek, Korea) and housed. A total of 48 mice were used after a 7-day acclimatization period. The mice were housed individually in polycarbonate cages under controlled environmental conditions (temperature: 23 ± 3°C, humidity: 55 ± 15%, light/dark cycle: 12 h, ventilation: 10–20 air changes per hour). The animals had free access to standard rodent chow and filtered water.

### Preparation of cell-free supernatants

Lactic acid bacteria (LAB) strains were cultured in de Man, Rogosa, and Sharpe (MRS) broth at 37°C under anoxic conditions for 16–20 h. For preparation of cell-free supernatants (CFS), 1 ml of each culture was centrifuged at 10 000 rpm for 1 min. The supernatant was collected and filtered through a 0.2 μm pore-size filter to obtain sterile CFS, which was used for antimicrobial assays.

### Antimicrobial activity of lactic acid bacteria cell-free supernatants

- Bacterial pathogens: *G. vaginalis* ATCC14018 and *Prevotella bivia* ATCC29303 were used as representative bacterial pathogens. Both strains were cultivated in modified Brain Heart Infusion medium (mBHI; BHI supplemented with 5% horse blood) at 37°C under anoxic conditions for 24–30 h. The pathogenic inocula were adjusted to 10^6^–10^7^ CFU/ml in mBHI. For each assay, 180 μl of the pathogen suspension was mixed with 20 μl of LAB CFS in a sterile 96-well plate. Control wells were prepared by mixing 180 μl of pathogen suspension with 20 μl of sterile MRS broth. All plates were incubated at 37°C under anoxic conditions for 24 h. Following incubation, bacterial viability was assessed by plating appropriate dilutions on chocolate agar plates. Colony-forming units (CFUs) were enumerated to determine the inhibitory effects of LAB CFS compared with controls.
- Fungal pathogen: *Candida albicans* ATCC10231 was used as a representative fungal pathogen. The strain was cultured in yeast–malt (YM) broth at 30°C under oxic conditions for 24–30 h. The inoculum was adjusted to 10^5^–10^6^ CFU/ml in YM broth. For each assay, 100 μl of the *C. albicans* ATCC10231 suspension was mixed with 100 μl of LAB CFS in a sterile 96-well plate. Control wells consisted of 100 μl of *C. albicans* ATCC10231 suspension and 100 μl of sterile MRS broth. Plates were incubated at 30°C under oxic conditions for 24 h. After incubation, viable yeast counts were determined by plating appropriate dilutions on YM agar plates, and CFUs were enumerated to evaluate antifungal activity relative to controls.

### Preparation of *Gardnerella vaginalis*

*Gardnerella vaginalis* was cultured in NYCIII broth containing NaCl, proteose peptone, yeast extract, and horse serum (Kisanbio, Seoul, Korea), along with HEPES buffer and glucose (Sigma-Aldrich, St. Louis, MO, USA) under anoxic conditions using anoxic jars (Kisanbio, Seoul, Korea) at 37°C for 48 h. The culture was then diluted to an optical density (OD_600_) of 0.5, and serial dilutions were plated onto NYCIII agar. Plates were incubated anaerobically at 37°C for 48 h, after which CFUs per milliliter (CFU/ml) were calculated.

### Time-dependent antimicrobial activity assay

The time-dependent antimicrobial activity of CFS derived from LAB was evaluated as previously described [21], with minor modifications. Antimicrobial effects were assessed against *G. vaginalis* ATCC 14018, *P. bivia* ATCC 29303, and *C. albicans* ATCC 10231. Pathogens were cultured under optimal conditions and adjusted to approximately 10^6^–10^7^ CFU/ml for *G. vaginalis* and *P. bivia*, and 10^5^ CFU/ml for *C. albicans*. CFS was prepared from cultures of *L. helveticus* 20838, *L. plantarum* ATCC 14917, and *L. rhamnosus* GG ATCC 53103 grown in MRS broth at 37°C for 24 h under facultative anoxic conditions. Supernatants were collected and sterilized by filtration through a 0.22-μm membrane filter. For bacterial assays, 9 ml of pathogen suspension was mixed with 1 ml of either CFS or MRS broth (negative control). For *C. albicans*, equal volumes (5 ml each) of yeast suspension and CFS or YM broth (negative control) were combined as described previously [22]. *Gardnerella vaginalis* and *P. bivia* were incubated anaerobically at 37°C in modified BHI broth supplemented with 5% (v/v) horse blood, whereas *C. albicans* was incubated aerobically at 30°C in YM broth. Samples were collected at 0, 4, 8, 12, 24, and 48 h, serially diluted, and plated for viable cell enumeration. *Gardnerella vaginalis* and *P. bivia* were plated on blood agar and incubated anaerobically at 37°C, whereas *C. albicans* was plated on YM agar and incubated aerobically at 30°C. Antimicrobial activity was determined based on time-dependent changes in viable counts relative to the corresponding negative controls. All experiments were performed in biological triplicate.

### Inhibition of biofilm formation

Biofilm inhibition was assessed using a crystal violet staining assay with minor modifications of a previously described method [23]. *Gardnerella vaginalis* ATCC 14018, *P. bivia* ATCC 29303, and *C. albicans* ATCC 10231 were used as representative vaginal pathogens. Each strain was cultured under optimal conditions and adjusted to 10^5^–10^6^ CFU/ml for *G. vaginalis* and *P. bivia*, and 10^5^ CFU/ml for *C. albicans*. Aliquots (100 μl) of each suspension were dispensed into 96-well microtiter plates. To evaluate inhibitory activity, 100 μl of CFS was added to treatment wells, whereas control wells received an equal volume of MRS broth (final volume: 200 μl per well). Plates containing *G. vaginalis* and *P. bivia* were incubated anaerobically at 37°C for 48 h in modified BHI (mBHI) supplemented with 5% (v/v) horse blood without agitation. *Candida albicans* plates were incubated aerobically at 30°C for 48 h in YM broth without agitation. Following incubation, planktonic cells were removed, and wells were gently washed twice with PBS. Biofilms were stained with 0.1% (w/v) crystal violet for 15 min at room temperature. After washing to remove excess stain, bound dye was solubilized in 200 μl absolute ethanol for 15 min, and absorbance was measured at 600 nm using a microplate reader. All experiments were performed in biological triplicate.

### Induction of bacterial vaginosis

To induce BV, the mice were subcutaneously pre-treated with β-estradiol-3-benzoate (Sigma-Aldrich, St. Louis, MO, USA) 72 h prior to bacterial inoculation. *Gardnerella vaginalis* was cultured and adjusted to a concentration of 1 × 10^8^ CFU in 20 μl and subsequently administered intravaginally on day 0.

### Administration of *Lactobacillus helveticus* 20838

*Lactobacillus helveticus* 20838 was administered orally (1 × 10^9^ CFU/200 μl) or intravaginally (1 × 10^8^ CFU/25 μl) once daily, starting either 5 days prior to (Day 5) or 1 day after (Day 1) the inoculation of *G. vaginalis*, depending on the experimental group. Treatment was continued for 13–19 consecutive days, after which necropsy was performed on the final day of the experiment.

### Histological analysis of vaginal epithelium

Formalin-fixed vaginal tissues were subjected to routine histological processing, including trimming, dehydration, paraffin embedding, and microtome sectioning (4 μm), followed by hematoxylin and eosin (H&E) staining. Staining was performed using an automated stainer (ST5010/CV5030; Leica Microsystems, Wetzlar, Germany). The staining procedure included deparaffinization in xylene, rehydration with graded ethanol solutions (DAEJUNG, Siheung, Korea), staining with Harris H&E Y (BBC, Mount Vernon, WA, USA), and mounting with a synthetic mountant (Thermo, Waltham, MA, USA). Histological analysis was performed using a light microscope (Axio Scope.A1; Zeiss, Oberkochen, Germany). Vaginal epithelial thickness was measured at three anatomical levels (proximal, middle, and distal) using Zen 3.4 software (Zeiss, Oberkochen, Germany), and the mean value was calculated from three measurements per region.

### Sample collection and processing at necropsy

At necropsy, the mice were anesthetized and euthanized. Vaginal fluid, vaginal tissue, and fecal samples were collected for analysis. Vaginal fluid was obtained by flushing the vaginal lumen 10 times with 50 μl of PBS (GenDPOT, Hanam, Korea) using a pipette, and the fluid was collected in microtubes. Vaginal tissues were bisected; one half was fixed in 10% neutral-buffered formalin (DAEJUNG, Siheung, Korea) for histological examination, and the other half was stored at −70°C (Thermo Scientific, Waltham, MA, USA) for RNA extraction and gene expression analysis. Fecal samples were stored at −70°C and subsequently used for gut microbiota profiling using 16S rRNA gene-based high-throughput sequencing. All animal procedures were conducted at the HLB BioStep animal facility (3rd animal housing unit, Korea) under SPF conditions. The study protocol was reviewed and approved by the Institutional Animal Care and Use Committee of HLB BioStep (IACUC No. BIOSTEP IACUC 23-KE-0509).

### Quantification of *Gardnerella vaginalis* colonization

To assess the colonization of *G. vaginalis* in the vaginal tract, vaginal fluid samples were subjected to 10-fold serial dilutions in phosphate-buffered saline (PBS; GenDPOT, Hanam, Korea). Diluted samples were plated onto NYCIII agar and incubated under anoxic conditions at 37°C for 48 h in an anoxic jar (Kisanbio, Seoul, Korea). Following incubation, CFUs were enumerated on plates containing 30–300 colonies, and the bacterial concentration was expressed as CFU per milliliter.

### Quantification of pro-inflammatory cytokines in vaginal tissue lysates

Total RNA was extracted from the vaginal tissues using TRIzol reagent (Thermo Scientific, Waltham, MA, USA) in pre-filled bead tubes and homogenized using Bead Ruptor Elite (Omni International, Kennesaw, GA, USA). RNA was isolated using chloroform and isopropanol, followed by ethanol washing and centrifugation at 13000 rpm for 5 min at 4°C. After discarding the supernatant, the RNA pellet was dissolved in DEPC water. RNA concentration and purity were measured using a NanoDrop 2000 spectrophotometer (Thermo Scientific, Waltham, MA, USA), and equal amounts of RNA were reverse-transcribed into complementary DNA (cDNA).

cDNA synthesis was performed using ReverTraAce qPCR RT Master Mix with gDNA Remover (Toyobo, Osaka, Japan) according to the manufacturer’s instructions. The thermal cycling protocol was as follows: 37°C for 15 min, 50°C for 5 min, 98°C for 5 min, followed by a hold at 4°C. Quantitative real-time PCR was performed using the CFX Connect Real-Time PCR Detection System (Bio-Rad, Hercules, CA, USA) to measure the expression levels of IL-1β, TNF-α, and GAPDH.

The primer sequences were as follows: IL-1β forward; 5′-TGTCTTGGCCGAGGACTAAGG-3′ and reverse 5′-TGGGCTGGACTGTTTCTAATGC-3′; TNF-α forward 5′-AGAAACACAAGATGCTGGGACAGT-3′ and reverse 5′-CCTTTGCAGAACTCAGGAATGG-3′; and GAPDH forward 5′-AAATGGTGAAGGTCGGTGTGAAC-3′ and reverse 5′-CAACAATCTCCACTTTGCCACTG-3′.

PCR amplification was conducted using qPCRBIO SyGreen Blue Mix Separate-Rox (PCR Biosystems, London, UK) according to the manufacturer’s instructions. The thermal cycling conditions were as follows: initial denaturation at 95°C for 2 min, followed by 40 cycles of 95°C for 5 s, 60°C for 30 s, and 65°C for 5 s, with a final denaturation step at 95°C for 5 s. Relative gene expression was calculated using the ${2}^{-\Delta{\Delta \mathrm{C}}_{\mathrm{T}}}$method with GAPDH as the reference gene.

### Microbiota profiling via 16S rRNA gene sequencing

Genomic DNA was extracted from vaginal fluid and fecal samples using the Mag-Bind Universal Pathogen Kit (Omega Bio-tek, Norcross, GA, USA), following the manufacturer’s protocol. Briefly, fecal pellets and vaginal fluid were suspended in 275 μl of SLX-Mlus Buffer and subjected to mechanical lysis using a MixerMill MM400 bead beater (Retsch, Haan, Germany). DNA was then isolated, purified, and eluted.

The hypervariable regions of the bacterial 16S rRNA gene were amplified using a forward primer (5′-TCGTCGGCAGCGTCAGATGTGTATAAGAGACAGCCTACGGGNGGCWGCAG-3′) and a reverse primer (5′-GTCTCGTGGGCTCGGAGATGTGTATAAGAGACAGGACTACHVGGGTATCTAATCC-3′).

PCR amplification was performed using 2× KAPA HiFi HotStart ReadyMix (Roche, Basel, Basel-Stadt, Switzerland), with an initial denaturation at 95°C for 3 min, followed by 25 cycles of 95°C for 30 s, 55°C for 30 s, and 72°C for 30 s, and a final extension at 72°C for 5 min.

PCR products were cleaned using HiAccuBeads (AccuGene, Incheon, South Korea) and a magnetic stand. Indexing PCR was then performed using IDT indexing primers (Integrated DNA technologies, Coralville, Iowa, US), 2× KAPA HiFi HotStart ReadyMix, and PCR-grade water. Cycling conditions were: 95°C for 3 min, followed by 8 cycles of 95°C for 30 s, 55°C for 30 s, and 72°C for 30 s, with a final extension at 72°C for 5 min and a hold at 4°C. After the clean-up step, the concentration of libraries was measured using a Qubit 4.0 fluorometer (Thermo Fisher Scientific, Waltham, MA, USA) with the 1× dsDNA High Sensitivity Assay Kit (Thermo Fisher Scientific, Waltham, MA, USA).

Raw paired-end 16S rRNA gene sequencing reads generated on the MiSeq System (Illumina) were initially demultiplexed based on unique sample-specific barcodes.

Downstream bioinformatics processing was performed using QIIME2 (v 2021.2) following standard workflows [24]. Primer trimming, quality filtering, denoising, paired-end read merging, and chimera removal were conducted using the DADA2 plugin (qiime dada2 denoise-paired), to infer amplicon sequence variants (ASVs). Specifically, forward and reverse reads were trimmed at 17 bp and 21 bp, respectively, to remove primer sequences. Based on per-base quality score profiles, reads were truncated at 270 bp (forward) and 220 bp (reverse) to retain high-quality sequences whereas minimizing error rates. All other DADA2 parameters were maintained at their default settings. Taxonomic classification of ASVs was performed using a naïve Bayes classifier trained on the SILVA reference database (version 138), amplified by the primers used in this study [25]. Taxonomic assignments were generated using the qiime feature-classifier classify-sklearn plugin with default confidence thresholds. The proportions of ASVs and reads assigned at the genus and species levels were calculated based on valid taxonomic annotations, excluding unassigned, unknown, and empty entries, along with their corresponding read counts. After DADA2 processing, samples with fewer than 15 000 reads were excluded from downstream analyses. As a result, one vaginal fluid sample in the GV + vLH group did not meet this sequencing depth criterion and was excluded, resulting in a final sample size of *n* = 7 for this group, whereas all other groups contained *n* = 8 samples. Alpha-diversity metrics (Observed, Shannon, and Simpson) were calculated using R packages phyloseq (v 1.46.0) on non-rarefied count data. For beta-diversity feature tables were rarefied to a minimum library size, and community dissimilarity was assessed using multiple distance metrics (Weighted UniFrac, Unweighted UniFrac, Bray-Curtis, and Jaccard) and visualized by principal coordinate analysis (PCoA). Differential abundance analyses were conducted using DESeq2 on non-rarefied count data, as described in the Statistical Analysis section.

Taxonomic assignments were considered reliable when supported by consistent classification confidence across samples. Given the limitations of 16S rRNA gene sequencing, genus-level assignments with low confidence or inconsistent prevalence were excluded from primary analyses, and higher taxonomic ranks were emphasized. Sequence similarity between *Lactobacillus*-associated ASVs and the 16S rRNA gene sequence of *L. helveticus* 20838 was evaluated using pairwise sequence alignment (Biostrings package in R). The reference sequence was compared with ASV sequences, and percent identity and alignment length were calculated based on the overlapping regions.

### 16S rRNA gene sequencing of *Lactobacillus helveticus* 20838

Pure cultures of *L. helveticus* 20838 were grown on MRS agar at 37°C for 24 h. The plate was sent to Macrogen Inc. (Seoul, South Korea) for bi-directional 16S rRNA gene sequencing. The assembled 16S rRNA gene sequence of *L. helveticus* 20838 was compared against reference sequences using BLASTn (Basic Local Alignment Search Tool for nucleotides) [26]. Sequence similarity searches were performed against the NCBI 16S ribosomal RNA sequences (Bacteria and Archaea) database, using default BLASTn parameters. Taxonomic assignment was based on highest sequence identity and alignment coverage. Bi-directional sequencing results were assembled using Codon Code Aligner (Codon Code Corporation, USA) and compared with reference sequences from the GenBank database (PX488011; https://www.ncbi.nlm.nih.gov/genbank/).

### Biogenic amine production assay

LABs were grown on the optimal growth, streaked out onto special medium with precursor of histamine, cadaverine, tyramine, and putrescine according to Bover-Cid and Holzapfel [27], and incubated for 4 days at 37°C. After incubation for 4 days, check the change in the color of the medium to determine the positive and negative. *Escherichia coli* ATCC 25922 was used as a positive control.

### Gelatine hydrolysis test

The basic protocol was followed according to the ASM Science Recommendation [28]. LABs grown under optimal growth conditions were inoculated with an inoculation loop into a gelatine medium and incubated at 30°C for up to 5 days and checked daily for gelatine liquefaction and bacterial growth. Gelatine normally liquefies at 28°C and above. To confirm that liquefaction was due to gelatinase activity, the tubes were immersed in a refrigerator for 30 min. Afterwards, tubes are tilted to observe if gelatine has been hydrolyzed. Hydrolysis of gelatine will result in a liquified medium even after exposure to cold temperature. *Bacillus cereus* ATCC 11778 was used as a positive control.

### Antibiotic resistance test

The basic protocol was based on ISO recommendation (ISO-10932, 2010). The broth dilution method was used to assess the minimal inhibitory concentration (MIC) of the strain against antibiotics. In the broth microdilution, test organisms were purely cultivated in broth media, and then all organisms were washed with 1× PBS. The bacterial solution washed in PBS was adjusted to 0.1–0.2 of 600 nm optical density (OD) units. 10 μl (1–3 × 107 CFU/ml) of the strain was inoculated in 96 well plates containing 19 μl of LSM broth media with antibiotics (approximately 1–3 × 104 CFU/well). The strain was considered susceptible when it was inhibited at a concentration of a specific antibiotic equal to or lower than the established cut-off value and resistant when it was not inhibited at a concentration of a specific antibiotic higher than the established cut-off value according to the parameters established by the European Food Safety Authority (EFSA, 2018).

### Whole genome sequencing of *Lactobacillus helveticus* 20838

Traditionally, DNA–DNA hybridization has served as the gold standard for bacterial species delineation since the 1960s [29]. However, with the advent of gene-based sequencing technologies, average nucleotide identity (ANI) has emerged as a more robust and widely accepted metric for assessing genomic similarity between prokaryotic strains [30]. The DNA of *L. helveticus* 20838 was extracted after full growth using MagListo Genomic DNA Extraction Kit (Qiagen) following the instructions of the manufacturer. SMRTbell DNA template libraries of 20-kb average insert size for the bacterial samples were prepared according to the manufacturer’s specification, with G-tubes (Covaris) used for fragmentation. SMRT sequencing was carried out on the PacBio RS II according to standard protocols, with the XL binding kit used in conjunction with the C4 sequencing kit. All runs were carried out with diffusion-based loading and analyzed using the standard primary data analysis. Raw PacBio long-read sequencing data were processed using the Hierarchical Genome Assembly Process (HGAP) pipeline for de novo assembly, followed by consensus polishing with Quiver. Genome annotation was performed using standard prokaryotic annotation workflows, and assembled sequences were used for downstream comparative genomic and safety analyses. Assembly quality metrics, genome statistics, and annotation results are summarized in Supplementary Tables S1–S3. Source codes for an implementation of HGAP and Quiver, data sets, and additional documentation are available at http://www.pacbiodevnet.com/HGAP and http://www.pacbiodevnet.com/quiver.

Putative virulence genes were screened using VirulenceFinder implemented via the Center for Genomic Epidemiology (CGE) web platform. The assembled whole-genome sequence of *L. helveticus* 20838 was queried against the VirulenceFinder database, which contains curated virulence-associated genes from clinically relevant bacterial pathogens. Searches were performed using default parameters, with a minimum sequence identity threshold of 90% and a minimum coverage threshold of 60% [31].

Acquired antimicrobial resistance genes were screened using ResFinder (version 4.1) implemented via the CGE web platform (https://www.genomicepidemiology.org/) [32]. The assembled whole-genome sequence of *Lactobacillus helveticus* 20838 was queried against the ResFinder database using default parameters, with a minimum sequence identity threshold of 90% and a minimum coverage threshold of 60%. Analyses were performed on 30 April 2025.

## Statistical analysis

Statistical analysis was performed using R software (version 4.3.0) [33]. The colony formation of *G. vaginalis*, PCR, and histological data were expressed as mean ± standard deviation (SD). Group differences were analyzed using two-way ANOVA followed by Dunnett’s multiple comparisons test. Results with *P*-values ≤.05 were considered statistically significant. For microbiome data, α-diversity metrics (Observed, Shannon, and Simpson) and rarefied count-based boxplots were compared using the Wilcoxon rank-sum test using the wilcox.test function in R. β-diversity (weighted UniFrac, unweighted UniFrac, Bray–Curtis, and Jaccard) was analyzed using PERMANOVA implemented via the adonis function in the vegan (v2.6.8) R package [34]. Differential abundance analysis was performed using DESeq2 (v1.42.1) implemented in R [35]. Raw ASV count tables (non-rarefied) were used as input, and library size normalization was conducted internally by DESeq2 using the median-of-ratios method. Count data were modeled using a negative binomial distribution, and log2 fold changes were estimated following variance stabilization. Taxa were considered differentially abundant based on predefined log2 fold-change and adjusted *P*-value thresholds, as described above. Given the compositional nature of microbiome data, DESeq2 results were interpreted conservatively as relative differential signals, and conclusions were supported by concordant changes observed in diversity metrics and taxonomic summaries.

## Results

### Characterization and safety evaluation of *Lactobacillus helveticus* 20838

LAB promotes host health through antimicrobial activity, immune modulation, and reinforcement of epithelial barriers. Newly isolated LAB strains intended for probiotic or biotechnological applications require rigorous safety evaluation to exclude transferable antibiotic resistance or virulence traits [36]. Regulatory authorities increasingly mandate evidence that such strains do not harbor transferable resistance to clinically relevant antibiotics, nor possess virulence-associated genes. This is especially critical for probiotics intended for direct human consumption, where strain-specific safety, including genomic stability and lack of pathogenicity, must be clearly demonstrated [37].

**Figure 1:**
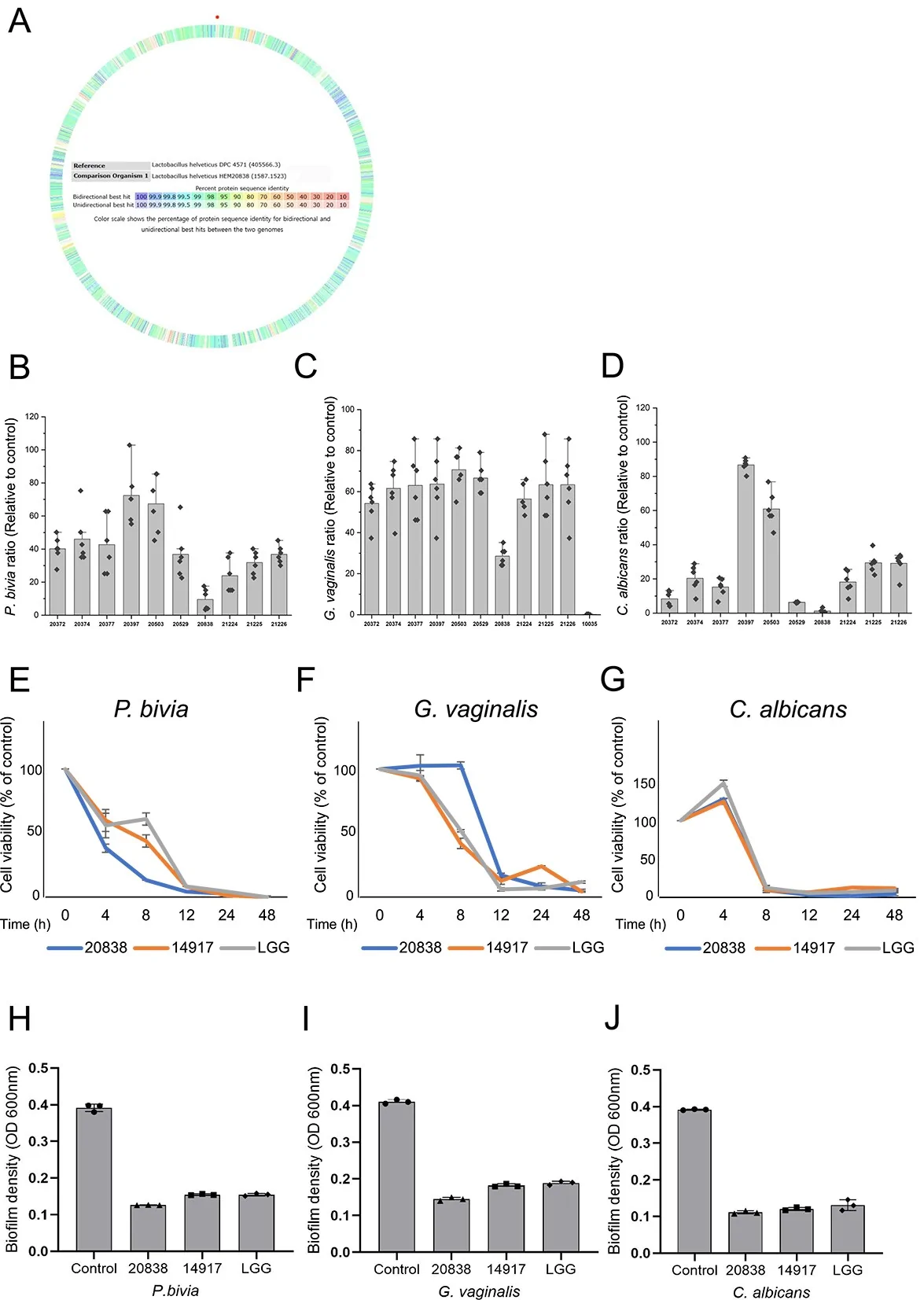
Characterization of *L. helveticus* 20838 and antimicrobial activity against pathogens. (A) Whole-genome comparative analysis of *L. helveticus* 20838 and the reference strain *L. helveticus* DPC4571. (B–D) Antimicrobial activity of *L. helveticus* 20838 and comparator LAB strains against representative vaginal pathogens. Viability assays were performed with (B) *P. bivia*, (C) *G. vaginalis*, and (D) *C. albicans* following co-incubation with LAB strains. (E–G) Time-dependent antimicrobial activity of CFS derived from LAB against vaginal pathogenic microorganisms. Relative viability of (E) *P. bivia*, (F) *G. vaginalis*, and (G) *C. albicans* was monitored at 0, 4, 8, 12, 24, and 48 h by plate counting. Data are expressed as relative survival (%) normalized to the initial cell count at 0 h (100%) and presented as mean ± SD (*n* = 3). (H–J). Biofilm formation by (H) *P. bivia* (PB), (I) *G. vaginalis* (GV), and (J) *C. albicans* (CA) was quantified. Data are presented as mean ± SD (*n* = 3). Statistical significance was determined by *t-*test compared with the LGG group (^**^P < .01, ^***^P < .001).

In the current study, we isolated a new LAB strain, *L. helveticus* 20838, for immune resistance against external stimulation and evaluated the phylogenetic relatedness of *L. helveticus* 20838. Its whole-genome sequence was compared to that of the well-characterized reference strain *L. helveticus* DPC4571. In the case of *L. helveticus* 20838, the ANI value was compared to that of *L. helveticus* DPC4571, a strain that has been widely used in this field and was calculated by the ANI algorithm [38] of the entire gene sequence; it showed 97.87% agreement. (Fig. 1A; Supplementary Tables S1–S3). To evaluate the safety and phenotypic characteristics of *L. helveticus* 20838, a series of tests was conducted in accordance with ISO guidelines (ISO 10932:2010), as detailed in Supplementary Tables S4 and S5. The 16S rRNA gene sequence of *L. helveticus* 20838 (Supplementary Table S6) was analyzed and confirmed to be taxonomically classified as *L. helveticus*, exhibiting high sequence identity with reference strains (Supplementary Table S7). Hemolytic activity testing revealed γ-hemolysis, indicating the absence of hemolytic activity and suggesting non-pathogenicity (Supplementary Table S8). The strain did not produce any of the four tested biogenic amines: histamine, cadaverine, tyramine, or putrescine, after 4 days of incubation at 37°C, in contrast to the positive control *E. coli* ATCC 25922, which showed positive production (Supplementary Table S9). Further safety evaluation showed that *L. helveticus* 20838 was negative for gelatinase activity, indicating an absence of proteolytic degradation, unlike the gelatinase-positive control (Supplementary Table S10). Antimicrobial susceptibility was assessed using the broth microdilution method. The strain was susceptible to all tested antibiotics, with minimum inhibitory concentrations (MICs) below or equal to the EFSA-established breakpoints for the *Lactobacillus* genus (Table 1). To further evaluate the safety profile of *L. helveticus* 20838, its whole-genome sequence was compared with those of representative pathogenic bacteria including *E. coli*, *Enterococcus spp.*, *Listeria monocytogenes*, and *Staphylococcus aureus*. Key virulence determinants such as *E. coli* Shiga toxins, *S. aureus* exoenzymes, and host immune evasion or toxin-related genes were not detected in *L. helveticus* 20838 (Supplementary Table S11). Additionally, genome-wide screening using the VirulenceFinder tool revealed no hits for major virulence genes (Supplementary Table S12). The strain was also subjected to antibiotic resistance profiling via the ResFinder platform, which confirmed the absence of known antibiotic resistance genes. Together, these data support the genomic stability and safety profile of *L. helveticus* 20838, indicating its suitability for probiotic development.

**Table 1:** Minimum inhibitory concentrations (MICs) of antibiotics for LABs.

	**Minimum inhibitory concentration (mg/l) of antibiotics**								
**Strain**	**AMP**	**ERY**	**GEN**	**TET**	**STR**	**CHL**	**CLI**	**KAN**	**VAN**
*L. jensenii* 20372	0.5	≤0.25	8	≤0.5	2	4	≤0.25	8	2
*L. jensenii* 20374	0.5	≤0.25	8	≤0.5	8	4	≤0.25	8	2
*L. jensenii* 20377	0.5	>0.25	>4	1	2	2	>0.25	8	2
*L. mellis* 20397	2	>0.25	>4	>0.25	2	2	>0.25	8	n.r
*L. melliventris* 20503	4	>0.25	>4	1	4	4	0.5	16	n.r
*L. coryniformis* 20529	0.5	≤0.25	2	8	2	4	≤0.25	32	n.r
*L. helveticus* 20838	0.5	≤0.25	4	1	2	4	≤0.25	16	≤0.5
*L. coryniformis* 21224	≤0.25	≤0.25	≤2	≤1	16	4	≤0.25	8	n.r
*L. coryniformis* 21225	1	≤0.25	≤4	≤4	≤4	4	≤0.50	64	n.r
*L. coryniformis* 21226	1	≤0.25	≤4	8	≤4	4	≤0.50	64	n.r
EFSA breakpoint (obligate homofermentative)	2	1	16	4	16	4	4	16	2
EFSA breakpoint (obligate heterofermentative)	2	1	16	8	64	4	4	64	n.r

Amp = Ampicillin, Ery = Erythromycin, Gen = Gentamicin, Tet = Tetracycline, Str = Streptomycin, Chl = Chloramphenicol, Cli = Clindamycin, Kan = Kanamycin, Van = Vancomycin, n.r = non required

### Antimicrobial activity of *Lactobacillus helveticus* 20838 against vaginal pathogens and anti-inflammatory effects in vivo

BV and vulvovaginal candidiasis are among the most prevalent vaginal infections, often associated with *G. vaginalis*, *P. bivia*, and *C. albicans*. Given the clinical relevance of LAB as probiotic alternatives for infection prevention, we assessed the antimicrobial activity of *L. helveticus* 20838 against these pathogens. A panel of LAB strains—*L. jensenii* (20 372, 20 374, 20 377), *L. mellis* (20397), *L. melliventris* (20503), and *L. coryniformis* (20 529, 21 224, 21 225, 21 226)—was tested for comparison (Table 2). Among all strains, *L. helveticus* 20838 demonstrated the strongest inhibitory activity, reducing the viability of *P. bivia* and *G. vaginalis* and suppressing the growth of *C. albicans* (Fig. 1B–D). These findings suggest that *L. helveticus* 20838 produces antimicrobial metabolites such as organic acids, hydrogen peroxide, or bacteriocin-like compounds. The time-dependent antimicrobial activity of LAB-derived CFS was evaluated against *G. vaginalis*, *P. bivia*, and *C. albicans*. All CFS preparations—*L. helveticus* 20838, *L. plantarum* ATCC14917, and *L. rhamnosus* GG (LGG)—reduced microbial viability over time, although inhibition kinetics varied by pathogen and strain (Fig. 1E–G). For *P. bivia*, *L. helveticus* 20838 showed the fastest inhibition, with marked suppression at 4–8 h, whereas *L. plantarum* and LGG displayed slower early-phase effects. By 12 h, viability was reduced in all groups, and cells were nearly undetectable at 24 h. For *G. vaginalis*, viability remained stable during 0–8 h, followed by a sharp decline at 8–12 h. Whereas all CFS treatments suppressed growth thereafter, *L. plantarum* showed transient regrowth at 24 h, whereas *L. helveticus* and LGG maintained sustained inhibition through 48 h. For *C. albicans*, a transient increase at 4 h was observed in all groups, particularly with LGG, but viability declined sharply by 8 h and remained low thereafter. Overall, LAB-derived CFS exerted broad antimicrobial activity against bacterial and fungal pathogens, with *L. helveticus* 20838 demonstrating comparatively faster and more sustained inhibitory kinetics.

**Table 2:** Hemolysin, biogenic amine production, and gelatinase by LAB strains.

**Strain**	**Hemolysin**	**His**	**Tyr**	**Put**	**Cad**	**Gelatinase**
*L. jensenii* 20372	ɣ	–	–	–	–	–
*L. jensenii* 20374	ɣ	–	–	–	–	–
*L. jensenii* 20377	α	–	–	–	–	–
*L. mellis* 20397	ɣ	–	–	–		–
*L. melliventris* 20503	α	–	–	–	–	–
*L. coryniformis* 20529	ɣ	–	–	–	–	–
*L. helveticus* 20838	ɣ	–	–	–	–	–
*L. coryniformis* 21224	α	–	–	–	–	–
*L. coryniformis* 21225	α	–	–	–	–	–
*L. coryniformis* 21226	α	–	–	–	–	–
*B. cereus* ATCC 27348	ß	n.a	n.a	n.a	n.a	n.a
*E. coli* ATCC 25922	n.a	+	+	+	+	n.a
*B. cereus* ATCC 11778	n.a	n.a	n.a	n.a	n.a	+

ɣ = gamma, α = alpha, ß = beta, His = histamine, Tyr = tyramine, Put = putrescin, Cad = cadaverine, n.a = not analyzed

The antibiofilm activity of LAB-derived CFS was assessed using a crystal violet assay (Fig. 1H–J). All CFS treatments reduced biofilm formation compared with the untreated control. Using *L. rhamnosus* GG (LGG) as a reference, CFS from *L. helveticus* 20838 demonstrated stronger antibiofilm effects against bacterial pathogens. For *P. bivia*, biofilm formation was significantly lower in the *L. helveticus* 20838 group (0.126 ± 0.001) than in the LGG group (0.155 ± 0.004; *P* < .01). Similarly, for *G. vaginalis*, *L. helveticus* 20838 significantly reduced biofilm biomass (0.145 ± 0.005) compared with LGG (0.189 ± 0.005; *P* < .001). In contrast, no significant difference between *L. helveticus* 20838 and LGG was observed for *C. albicans*. *Lactobacillus plantarum* ATCC14917 showed no significant differences relative to LGG across all pathogens tested. Collectively, these findings indicate that *L. helveticus* 20838-derived CFS exhibits comparatively stronger antibiofilm activity against *P. bivia* and *G. vaginalis*.

The genomic analysis of *L. helveticus* 20838 revealed unique genes potentially contributing to its antimicrobial activity, including PGF_10330198 (NADH peroxidase) and PGF_076554591 (hydroperoxide resistance protein), among others (Supplementary Dataset S1). These genes may enhance oxidative stress resistance and antimicrobial metabolite production, supporting the observed inhibition of *G. vaginalis*, *P. bivia*, and *C. albicans*. This dual inhibitory effect highlights the clinical relevance of *L. helveticus* 20838, as mixed infections involving BV-associated bacteria and *C. albicans* are frequently observed in gynecological practice.

**Figure 2:**
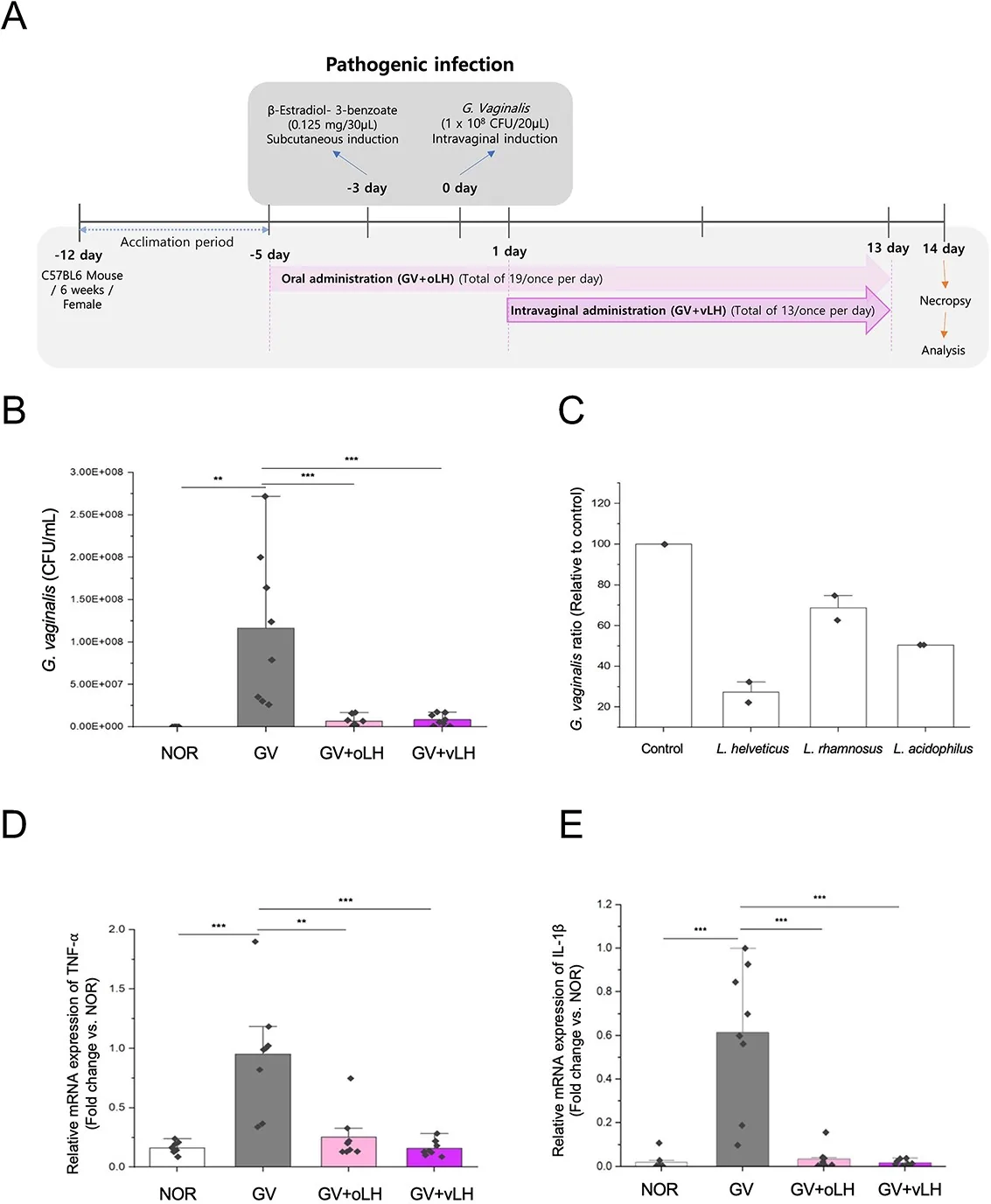
*L. helveticus* 20838 attenuates *G. vaginalis* proliferation and inflammatory gene expression in a murine vaginitis model. (A) Schematic illustration of the experimental design using C57BL/6 female mice infected with *G. vaginalis* and treated with *L. helveticus* 20838. (B) Colony formation of *G. vaginalis* in the NOR (normal), GV (*G. vaginalis*), GV + oLH (*G. vaginalis*+ oral *L. helveticus* 20838), and GV + vLH (*G. vaginalis*+ intravaginal *L. helveticus* 20838) groups. (C) Comparison of *G. vaginalis* proliferation following treatment with *L. helveticus* 20838 versus *L. rhamnosus* or *L. acidophilus*. (D–E) Relative mRNA expression levels of *Tnf-α* (D) and *Il-1β* (E) in vaginal tissues. Data are presented as mean ± s.d. ^***^P < .001 compared with GV; ^###^*P* < .001 compared with NOR (two-way ANOVA with Dunnett’s multiple comparison test).

To confirm these effects in vivo, we evaluated the efficacy of *L. helveticus* 20838 in a murine model of *G. vaginalis*-induced vaginitis. Six-week-old female C57BL/6 mice were acclimated for one week before infection. BV was induced by intravaginal inoculation of *G. vaginalis* (1 × 10^8^ CFU/20 μl). Mice were randomized into four groups: NOR (untreated controls; *n* = 8), GV (*G. vaginalis*-infected controls; *n* = 8), GV + oLH (*G. vaginalis* + oral *L. helveticus* 20838; *n* = 8), and GV + vLH (*G. vaginalis* + intravaginal *L. helveticus* 20838; *n* = 8). Probiotic treatment was administered once daily for 13 days, after which mice were sacrificed for analysis (Fig. 2A). Compared with the GV group, both oral and intravaginal administration of *L. helveticus* 20838 significantly reduced *G. vaginalis* burden (Fig. 2B). As depicted, *L. helveticus* 20838 achieved over a two-fold greater reduction in bacterial load compared with commonly used probiotics *Lactobacillus rhamnosus* and *Lactobacillus acidophilus* (Fig. 2C). In infected mice, vaginal tissues showed elevated expression of pro-inflammatory cytokines TNF-α and IL-1β relative to NOR controls. Treatment with *L. helveticus* 20838 suppressed these inflammatory markers, with intravaginal administration exerting slightly stronger effects than oral dosing, suggesting enhanced local bioavailability (Fig. 2D–E). These results demonstrate that *L. helveticus* 20838 is effective in reducing the number of *G. vaginalis* colonizing the vaginal tract in this experimental model. Together, these findings demonstrate that *L. helveticus* 20838 not only suppresses *G. vaginalis* colonization but also attenuates the associated inflammatory response, supporting its therapeutic potential for BV and related vaginal infections.

#### Lactobacillus helveticus 20838 prevents vaginal epithelial thickening induced by Gardnerella vaginalis

**Figure 3:**
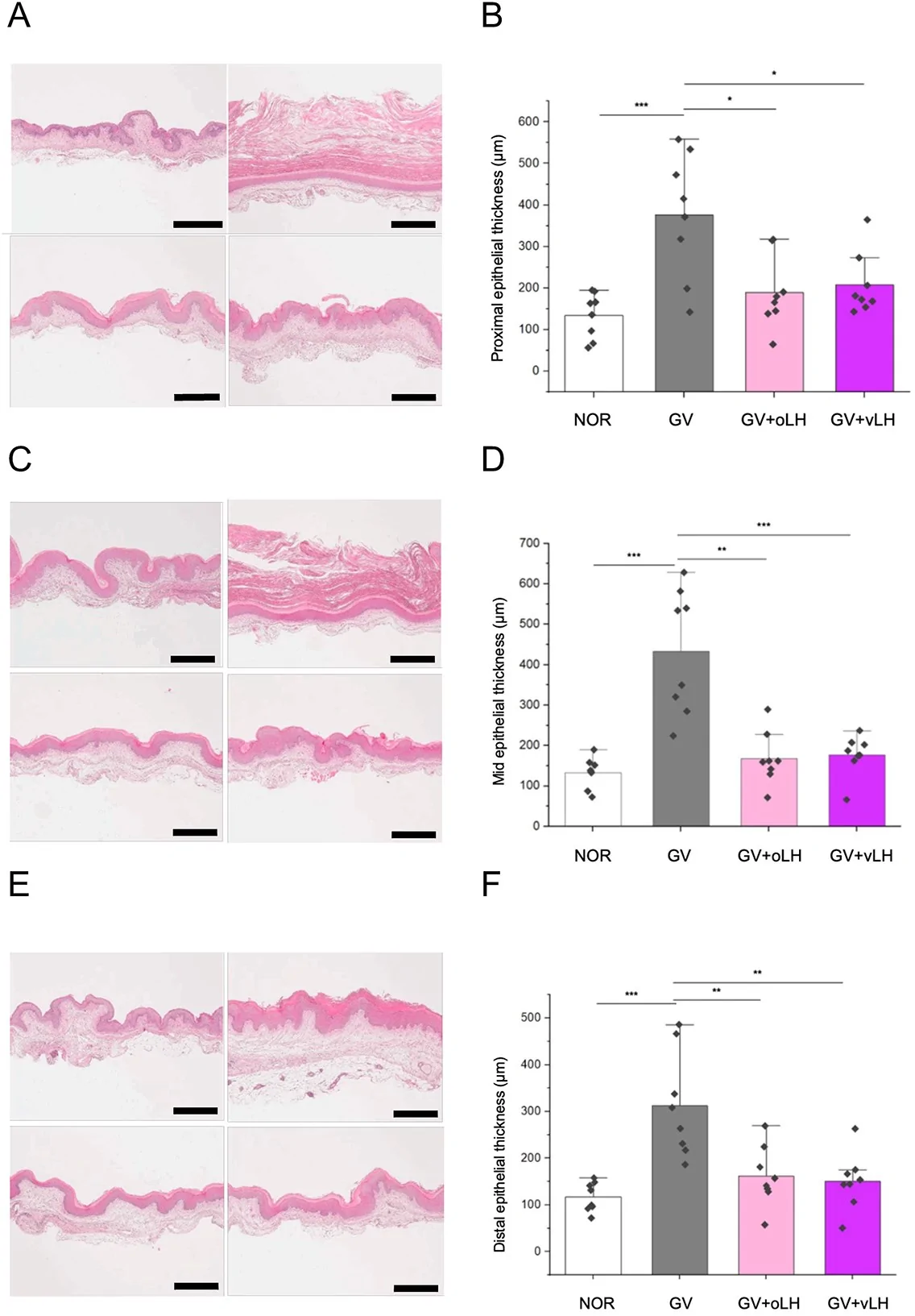
*L. helveticus* 20838 suppresses thickening on the vaginal wall due to vaginitis induced by *G. vaginalis*. (A) A H&E staining of the proximal vaginal wall of female mice with or without *L. helveticus* 20838 treatment. (B) The thickness of the proximal vaginal wall layers of the skin on the vagina of female mice (*N* = 8). (C) A H&E staining of middle vaginal wall of mice with or without *L. helveticus* 20838 injection. (D) The thickness of the mid-vaginal wall of the skin on the vagina (*N* = 8). (E) Histopathological examination of distal vaginal wall by H&E staining. (F) Distal vaginal wall thickness was measured (*N* = 8). Vaginal epithelium thickness was measured over time. The scale bar represents 50 μm. Data are presented as the mean ± s.d. ^###^*P* < .001 vs NOR group and ^*^P < .05, ^**^P < .01, and ^***^P < .001 vs GV group (two-way ANOVA, Dunnett’s multiple comparison).

Epithelial exfoliation and hyperplasia are characteristic histological features of BV. To evaluate whether *L. helveticus* 20838 could mitigate these alterations, H&E staining was performed to assess vaginal wall thickness. In the GV group, infection with *G. vaginalis* resulted in marked epithelial cell exfoliation and increased thickness of the stratum corneum and transitional epithelium. In contrast, both oral and intravaginal administration of *L. helveticus* 20838 substantially attenuated these pathological changes, restoring epithelial morphology to levels comparable to the NOR group. Regional analysis revealed significant thickening and hyperplasia in the proximal (Fig. 3A–B), middle (Fig. 3C–D), and distal (Fig. 3E–F) vaginal wall of the GV group. *Lactobacillus helveticus* 20838 treatment effectively normalized epithelial structure across all regions. Compared with the GV group, treatment with *Lactobacillus helveticus* 20838 resulted in a significant reduction in *G. vaginalis* burden (Kruskal–Wallis test followed by pairwise Wilcoxon rank-sum tests with Bonferroni correction, *P* < .001), indicating robust histological protection. Collectively, these findings demonstrate that *L. helveticus* 20838 counteracts *G. vaginalis*-induced epithelial remodeling and preserves vaginal tissue integrity under conditions of dysbiosis-driven inflammation.

#### *Lactobacillus helveticus* 20838 restores microbial diversity in the gut of vaginitis-induced mice

**Figure 4:**
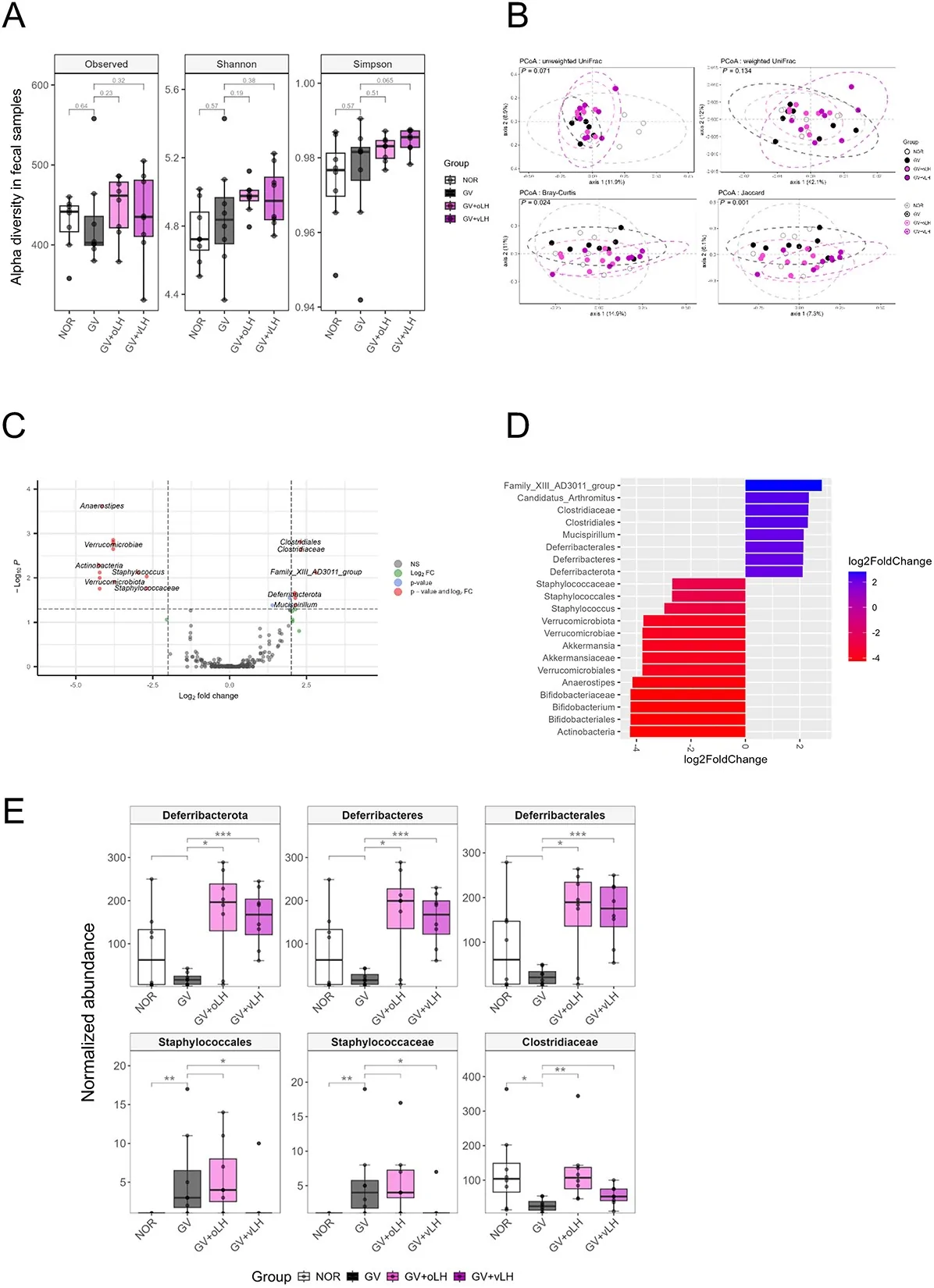
Comprehensive assessment for microbiome of fecal samples of female mice along with *L. helveticus* 20838 intervention. (A) Alpha-diversity measure of feces microbiome for each condition, NOR, GV, GV + oLH, or GV + vLH. (B) Beta-diversity of female mice was measured with or without *L. helveticus* 20838 application under vaginitis. (C) Enhanced volcano plot of significantly altered bacterial taxa in feces by *G. vaginalis* infection compared to NOR with log2 fold change ≥ |2| and *P*-value <.05 by DESeq2 analysis. (D) Bacterial taxa list which altered with vaginitis in fecal sample. (E) Normalized counts of differentially abundant taxa identified by DESeq2 analysis in fecal sample. *P*-values are listed in panel or ^*^P < .05, ^**^P < .01, and ^***^P < .001 (Wilcoxon rank-sum test).

To evaluate whether *G. vaginalis*-induced vaginitis disrupts systemic microbial homeostasis, microbial diversity in fecal and vaginal samples was analyzed. Alpha-diversity metrics revealed a significant reduction in microbial richness and evenness in the GV group compared with the NOR group, consistent with gut dysbiosis during vaginitis. Both oral and intravaginal administration of *L. helveticus* 20838 significantly restored alpha diversity, as evidenced by increased Shannon diversity index (GV + oLH: median 4.98; GV + vLH: median 4.95 vs. GV: median 4.84) (Fig. 4A). Beta-diversity further demonstrated distinct clustering between the GV and NOR groups, whereas *L. helveticus* 20838-treated mice exhibited microbiota profiles closely resembling those of healthy controls (Fig. 4B). These findings indicate that *G. vaginalis* infection perturbs the gut microbiota despite being localized to the vaginal mucosa, suggesting host–microbiome crosstalk along the gut–vagina axis. Differential taxonomic analysis revealed significant alterations in microbial composition in GV mice (Fig. 4C), many of which were normalized following *L. helveticus* 20838 treatment (Fig. 4D) (Supplementary Dataset S2). *Lactobacillus helveticus* 20838 promoted enrichment of beneficial commensal bacteria and suppressed taxa associated with BV pathogenesis (Fig. 4E). In particular, oral and intravaginal administration increased the abundance of *Deferribacterota*, *Deferribacteres*, *Deferribacterales*, and *Clostridiaceae*, whereas *Staphylococcales* and *Staphylococcaceae* were modestly elevated in the oral group but fully normalized in the intravaginal group. Due to limited and inconsistent genus-level classification confidence, differential abundance analyses were primarily interpreted at the family, order, and class levels. In the ASV table generated from the DADA2/QIIME2 pipeline, 65.7% of ASVs were assigned to the genus level and 44.9% to the species level. In terms of abundance, these corresponded to 95.7% and 74.1% of total reads, respectively. Although a substantial proportion of reads could be classified at the species level, we focused on genus-level interpretation to ensure robustness, given the known limitations in species-level resolution of the 16S rRNA. Together, these results demonstrate that *L. helveticus* 20838 not only alleviates local vaginal pathology but also counteracts vaginitis-associated dysbiosis in the gut, underscoring its dual role in maintaining microbial balance across mucosal sites.

#### *L. helveticus* 20838 modulates the vaginal microbiome composition in vaginitis-induced mice

**Figure 5:**
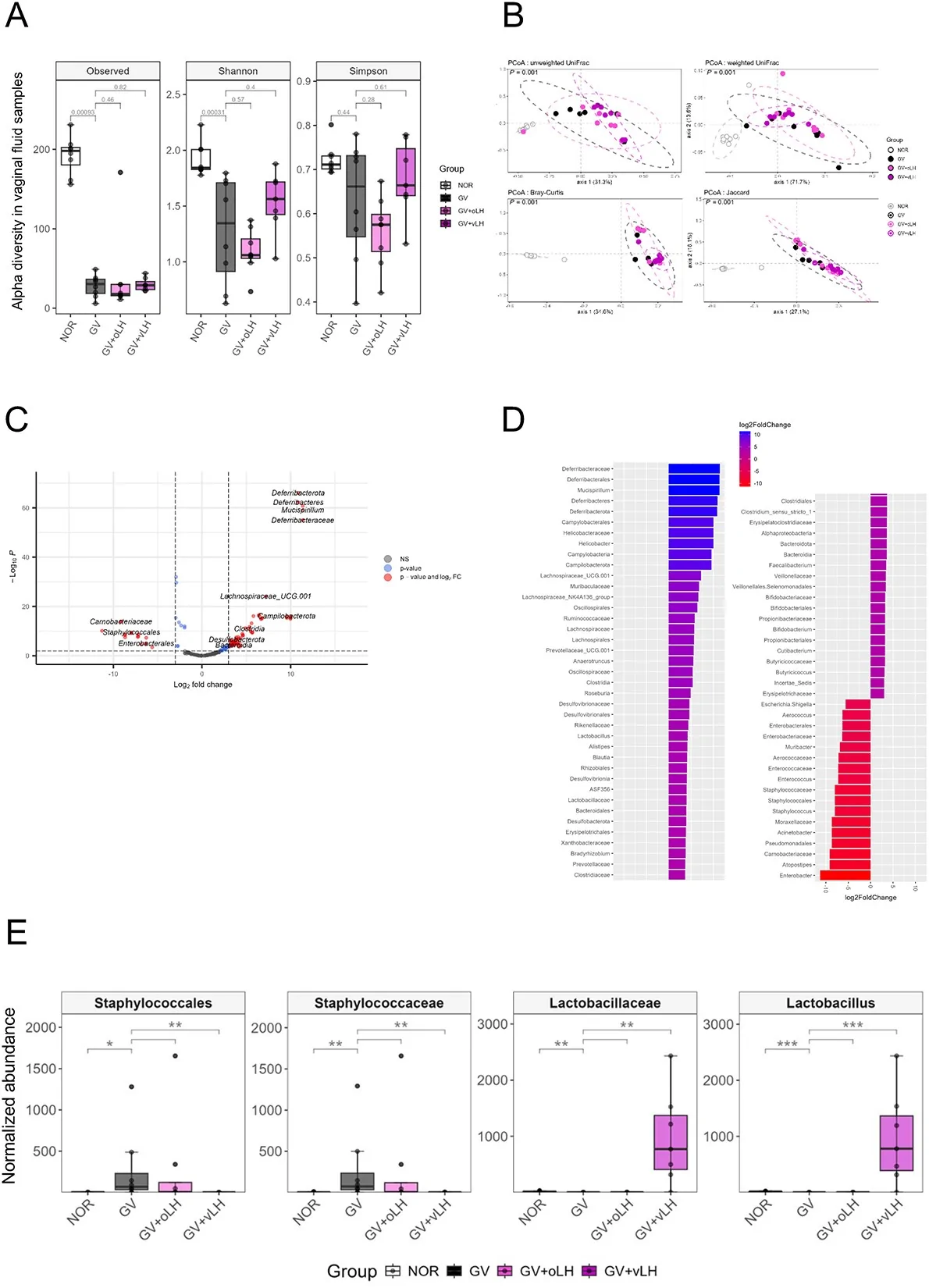
The impact of *L. helveticus* 20838 intervention with vaginitis for microbiome of vaginal fluid samples of female mice. (A) Alpha-diversity determines the vaginal fluid sample microbiome for each condition, NOR, GV, GV + oLH, or GV + vLH. (B) Beta-diversity of vaginal fluid samples was measured with or without *L. helveticus* 20838 applications under vaginitis. (C) Enhanced volcano plot of significantly altered bacterial taxa in vaginal fluid by *G. vaginalis* infection compared to NOR with log2 fold change ≥ |3| and *P*-value <.01 by DESeq2 analysis. (D) Bacterial taxa list which altered with vaginitis in vaginal fluid samples. (E) Normalized counts of differentially abundant taxa identified by DESeq2 analysis in vaginal fluid sample. *P*-values are listed in panel or ^*^P < .05, ^**^P < .01, and ^***^P < .001 (Wilcoxon rank-sum test).

To assess the local effects of *L. helveticus* 20838 on the vaginal microbiota, we analyzed microbial diversity in vaginal fluid samples. Consistent with observations in fecal samples, alpha-diversity indices showed a significant reduction in microbial richness and evenness in the GV group, as reflected by decreased Shannon diversity index (median 1.35 vs. 1.84 in NOR; *P* = .00031; Wilcoxon rank-sum test) (Fig. 5A). Beta-diversity analysis revealed clear separation between the GV and NOR groups, whereas the microbiota of *L. helveticus* 20838-treated mice clustered closer to healthy controls (Fig. 5B). Differential taxonomic analysis showed pronounced shifts in bacterial composition after *G. vaginalis* infection, with enrichment of dysbiosis-associated taxa (Fig. 5C–D) (Supplementary Dataset S2). *Lactobacillus helveticus* 20838 treatment reversed these alterations, increasing the relative abundance of beneficial lactobacilli, whereas reducing the prevalence of pathogenic taxa linked to vaginitis (Fig. 5E). As a result, *Lactobacillaceae* and *Lactobacillus* abundance were enriched only in the intravaginal *L. helveticus* 20838 group, suggesting superior local efficacy compared with oral administration. To further assess whether this enrichment was compatible with the administered strain, sequence similarity analysis was performed. Among the dominant *Lactobacillus*-associated ASVs identified within the top 20 taxa (Supplementary Table S3), one ASV showed 100% identity to the *L. helveticus* 20838. Although this ASV was assigned only at the genus level, the exact sequence match supports that the observed enrichment is compatible with the administered strain. These results indicate that *L. helveticus* 20838 plays a key role in re-establishing vaginal microbial balance, suppressing pathogenic taxa and promoting beneficial commensals, thereby contributing to restoration of vaginal homeostasis during dysbiosis.

## Discussion

The marked reduction in *G. vaginalis* colonization following *L. helveticus* 20838 administration underscores its strong antagonistic capacity. Consistent with previous reports of lactobacilli exerting pathogen inhibition through competitive exclusion, lactic acid production, and bacteriocin secretion [39, 40], *L. helveticus* 20838 outperformed commonly used probiotics such as *L. rhamnosus* and *L. acidophilus*. Its superior efficacy may reflect strain-specific attributes, including enhanced adhesion, robust acidification, and metabolic versatility under vaginal pH and oxidative stress [41]. Both oral and intravaginal administration reduced pathogen load; however, the intravaginal route produced stronger local effects, particularly the re-establishment of dominant lactobacilli, highlighting the importance of delivery strategy for probiotic efficacy [42, 43]*.* Additionally, its superior efficacy compared to commonly used probiotics, *L. rhamnosus* and *L. acidophilus*, suggests strain-specific mechanisms, such as enhanced adhesion properties and superior metabolic activity, which have been reported in other probiotic strains [44]. Although oral administration of vaginal probiotics has been proposed as a non-invasive intervention strategy, accumulating clinical evidence indicates that its efficacy in treating or preventing BV is limited and inconsistent. In particular, oral delivery often fails to achieve sustained vaginal colonization by lactobacilli and shows minimal impact on recurrence rates compared with local therapies. Recent clinical studies have highlighted that oral probiotic supplementation rarely leads to durable restoration of a lactobacilli-dominated vaginal microbiome, underscoring the challenge of translating gut-mediated effects into meaningful vaginal ecological changes [45]. Consistent with these clinical observations, oral administration of *L. helveticus* 20838 in the present study resulted in only modest modulation of host inflammatory responses and microbial profiles, whereas intravaginal administration produced more pronounced local effects. These findings support the interpretation that oral delivery may act, at best, as an adjunctive or indirect modulator—potentially via systemic immune or gut–vagina axis–mediated mechanisms—rather than as a stand-alone therapeutic approach for vaginal dysbiosis. Together, our results reinforce the clinical consensus that local administration is likely required for robust and reproducible vaginal microbiome modulation, whereas oral probiotics alone are unlikely to provide sufficient efficacy in clinical settings.

Inflammation is a central feature of BV, contributing to epithelial disruption and increased vulnerability to secondary infections [46]. In our model, *G. vaginalis* infection elevated *Tnf-α* and *Il-1β* expression, which was significantly reversed by *L. helveticus* 20838. These anti-inflammatory effects may be mediated through modulation of innate immune signaling, such as Toll-like receptor interference, or by microbial metabolites including short-chain fatty acids [47, 48]*.* In parallel, histological analysis showed that *L. helveticus* 20838 prevented pathological epithelial thickening, supporting its ability to protect mucosal integrity and counteract inflammation-driven tissue remodeling.

Microbiome analysis revealed profound dysbiosis in both vaginal and fecal samples of infected mice, which was significantly reversed by *L. helveticus* 20838 treatment. Microbiome profiling revealed that *L. helveticus* 20838 effectively restored both alpha and beta diversity disrupted by *G. vaginalis* infection. Differential taxonomic analyses showed suppression of dysbiosis-associated taxa and enrichment of beneficial commensals [49], particularly *Lactobacillus* spp. Intravaginal administration selectively enriched *Lactobacillus* abundance at the infection site, emphasizing the role of local colonization in therapeutic benefit. Beyond the vaginal niche, fecal analyses revealed that *G. vaginalis* infection induced systemic dysbiosis, with *L. helveticus* 20838 restoring microbial diversity and composition. In fecal samples, *L. helveticus* 20838 increased the relative abundance of *Deferribacterota*, *Deferribacteres*, *Deferribacterales*, and *Clostridiaceae*, whereas reducing overrepresented pathogenic taxa. As a result, *Staphylococcales* and *Staphylococcaceae* were slightly elevated after oral treatment but normalized in the intravaginal group, suggesting delivery route–specific effects on microbial composition. In vaginal samples, *L. helveticus* 20838 treatment restored alpha- and beta-diversity and led to a marked enrichment of *Lactobacillaceae* and *Lactobacillus* spp., which were absent in the *G. vaginalis*–infected group. These taxa shifts strongly indicate ecological rebalancing, favoring beneficial commensals and suppressing dysbiosis-associated organisms.

The dual restoration of gut and vaginal microbiota underscores systemic crosstalk along the gut–vagina axis, suggesting that oral probiotics may exert remote immunological and microbial effects, whereas intravaginal delivery maximizes local colonization. This dual mechanism supports a flexible therapeutic model, where *L. helveticus* 20838 could be formulated for both systemic and topical use. The observed strain-specific benefits over conventional probiotics reinforce the need for precise selection in probiotic development. Altogether, these findings position *L. helveticus* 20838 as a next-generation probiotic that combines pathogen suppression, immune modulation, and microbiome restoration. Its ability to enrich protective lactobacilli whereas reducing dysbiosis-associated taxa highlights its promise for clinical development in the prevention and management of recurrent BV and mixed vaginal infections.

Although clinical trials remain the gold standard for establishing therapeutic efficacy, experimental animal models provide indispensable advantages for mechanistic and ecological investigations that cannot be readily achieved in human cohorts. In particular, the murine model used in this study enables controlled interrogation of the gut–vagina axis, a central focus of our work, by allowing simultaneous and longitudinal assessment of microbial dynamics across two anatomically and ecologically distinct niches under strictly regulated conditions, including standardized diet, housing, and SPF environments. Such experimental control is not feasible in clinical studies, where inter-individual variability, uncontrolled lifestyle factors, and ethical constraints limit direct causal inference. Within this framework, the present model is not intended to recapitulate the full clinical complexity of human vaginitis or BV, but rather to serve as a reductionist and hypothesis-generating system to examine how localized vaginal perturbation can influence distal gut microbial communities, and how different routes of probiotic administration shape niche-specific and systemic responses. Accordingly, our findings provide mechanistic insight into delivery route–dependent effects and gut–vagina microbial crosstalk, whereas underscoring the need for subsequent human studies to validate translational relevance.

### Limitations of the study

Despite these promising findings, several limitations must be considered. First, although murine models provide valuable insights, they may not fully replicate the complexity of the human vaginal microbiome and immune responses. Second, the long-term colonization potential of *L. helveticus* 20838 and its sustained effects require further investigation. Third, even though we compared efficacy with representative probiotic strains, an in-depth analysis of genomic features, metabolomic profiling, and interaction with host epithelial signaling pathways is needed to elucidate the mechanistic underpinnings of its therapeutic action. Fourth, although our results suggest immunomodulatory and antimicrobial properties, the precise molecular mechanisms underlying these effects remain to be elucidated, and we represent that compositional methods such as ANCOM-BC2 provide an alternative framework for differential abundance testing, and future studies will benefit from applying these approaches in larger cohorts. Although the antimicrobial activity observed here is supported by time-kill kinetics and biofilm suppression assays, the precise molecular mediators remain to be defined. Ongoing work is focused on distinguishing acid-mediated effects from proteinaceous antimicrobial factors, including potential bacteriocins, through pH-neutralization, enzymatic degradation assays, and comparative genomic analysis. These studies will further elucidate the strain-specific mechanisms underlying pathogen suppression.

Although differential abundance was primarily interpreted at the family level due to limitations in genus-level classification confidence, the Clostridiaceae family includes several genera reported to exert immunomodulatory effects, including regulation of T-cell responses. Future studies employing higher-resolution sequencing approaches will be required to resolve genus- and species-specific contributors to these effects. Additionally, an important limitation of this study is the absence of baseline vaginal microbiome samples prior to *G. vaginalis* challenge and probiotic intervention. As a result, it is not possible to determine whether *Lactobacillus helveticus* 20838 directly reshapes the vaginal microbial community or primarily promotes restoration of taxa present before dysbiosis. Future studies incorporating longitudinal sampling, including pre-infection baseline profiles, will be essential to distinguish direct colonization effects from recovery-driven microbial reassembly.

## Supplementary Material

Supplementary_material_wrag118

## Data Availability

All data supporting the findings of this study are available within the article and its supplementary materials. The 16S rRNA gene sequencing of *L. helveticus* 20838, obtained by Sanger sequencing, has been deposited in GenBank under accession number PX488011. The whole-genome sequencing raw reads of *L. helveticus* 20838 have been deposited in the NCBI Sequence Read Archive (SRA) under BioProject accession number PRJNA1415474 (SRA accession: SRR37226682). The raw 16S rRNA gene sequencing data (FASTQ files) generated in this study have been deposited in the NCBI Sequence Read Archive (SRA) under BioProject accession number PRJNA1431109 (SRA accession: SUB15991229). Processed microbiome data, including ASV abundance tables and taxonomic assignments, are provided as Supplementary Data. Additional non-sequencing data supporting the findings of this study are available via Figshare at 10.6084/m9.figshare.30209176.
