# Correction to: Systems-level restoration of vaginal and gut microbiota by *Lactobacillus helveticus* 20838 alleviates *Gardnerella vaginalis*-induced dysbiosis

**DOI:** 10.1093/ismejo/wrag264

**Published:** 2026-10-24

This is a **correction** to:

Juewon Kim, Woo-il Kim, Kyuyeon Lee, Nayeon Kim, Ah Young Hwang, Dong Ho Suh, Donghyun Cho, Yosep Ji, Hye-Ji Kang, Eun Sung Jung, Systems-level restoration of vaginal and gut microbiota by *Lactobacillus helveticus* 20838 alleviates *Gardnerella vaginalis*-induced dysbiosis, *The ISME Journal*, Volume 20, Issue 1, January 2026, wrag118, https://doi.org/10.1093/ismejo/wrag118

In the originally published version of this manuscript, only panel A is displayed in Figure 1-5. Each Figure is erroneously missing the remainder of their panel.

This error has been corrected, and Figure 1-5 has been updated.

