# Supplementary material for: Systems-level restoration of vaginal and gut microbiota by Lactobacillus helveticus 20838 alleviates Gardnerella vaginalis-induced dysbiosis
Source: ISME J. 2026 May 17;20(1):wrag118. doi: 10.1093/ismejo/wrag118 (PMC13245722; doi:10.1093/ismejo/wrag118)
Supplement: Supplementary_material_wrag118 [file supplementary_material_wrag118.zip › Kim_SM.docx]

**Supplementary material**

Systems-level restoration of vaginal and gut microbiota by Lactobacillus helveticus 20838 alleviates Gardnerella vaginalis-induced dysbiosis

**Kim J. et al.**

**Supplementary methods**

**Tables S1-S12**

**Materials and methods**

**Growth conditions of LAB strains**

The growth conditions of lactic acid bacteria (LAB) strains were established in accordance with the International Organization for Standardization (ISO) recommendations (ISO 10932:2010, Supplement 1). *Lactobacillus helveticus* 20838; *Lactobacillus jensenii* 20372, 20374, and 20377; *Lactobacillus coryniformis* 20529, 21224, 21225, and 21226; *Lactobacillus mellis* 20397; and *Lactobacillus melliventris* 20503 were cultured in MRS broth at 37°C for 24 hours under anaerobic conditions.

**Preparation of Cell-Free Supernatants**

Lactic acid bacteria (LAB) strains were cultured in de Man, Rogosa, and Sharpe (MRS) broth at 37°C under anaerobic conditions for 16–20 h. For preparation of cell-free supernatants (CFS), 1 ml of each culture was centrifuged at 10,000 rpm for 1 min. The supernatant was collected and filtered through a 0.2 µm pore-size filter to obtain sterile CFS, which was used for antimicrobial assays.

**Antimicrobial Activity of LAB Cell-Free Supernatants**

(i) Bacterial Pathogens: *Gardnerella vaginalis* ATCC14018 and *Prevotella bivia* ATCC29303 were used as representative bacterial pathogens. Both strains were cultivated in modified Brain Heart Infusion medium (mBHI; BHI supplemented with 5% horse blood) at 37°C under anaerobic conditions for 24–30 h. The pathogenic inocula were adjusted to 10^6^–10^7^ CFU/ml in mBHI. For each assay, 180 µl of the pathogen suspension was mixed with 20 µl of LAB CFS in a sterile 96-well plate. Control wells were prepared by mixing 180 µl of pathogen suspension with 20 µl of sterile MRS broth. All plates were incubated at 37°C under anaerobic conditions for 24 h. Following incubation, bacterial viability was assessed by plating appropriate dilutions on chocolate agar plates. Colony-forming units (CFU) were enumerated to determine the inhibitory effects of LAB CFS compared with controls.

(ii) Fungal Pathogen: *Candida albicans* ATCC10231 was used as a representative fungal pathogen. The strain was cultured in yeast–malt (YM) broth at 30 °C under aerobic conditions for 24–30 h. The inoculum was adjusted to 10^5^–10^6^ CFU/ml in YM broth. For each assay, 100 µl of the *C. albicans* ATCC10231 suspension was mixed with 100 µl of LAB CFS in a sterile 96-well plate. Control wells consisted of 100 µl of *C. albicans* ATCC10231 suspension and 100 µl of sterile MRS broth. Plates were incubated at 30°C under aerobic conditions for 24 h. After incubation, viable yeast counts were determined by plating appropriate dilutions on YM agar plates, and CFU were enumerated to evaluate antifungal activity relative to controls.

**Time-Dependent Antimicrobial Activity Assay**

Antimicrobial effects were assessed against G. vaginalis ATCC 14018, P. bivia ATCC 29303, and C. albicans ATCC 10231. Pathogens were cultured under optimal conditions and adjusted to approximately 10⁶–10⁷ CFU/mL for G. vaginalis and P. bivia, and 10⁵ CFU/mL for C. albicans. CFS was prepared from cultures of L. helveticus 20838, L. plantarum ATCC 14917, and L. rhamnosus GG ATCC 53103 grown in MRS broth at 37°C for 24 h under facultative anaerobic conditions. Supernatants were collected and sterilized by filtration through a 0.22-µm membrane filter. For bacterial assays, 9 mL of pathogen suspension was mixed with 1 mL of either CFS or MRS broth (negative control). For C. albicans, equal volumes (5 mL each) of yeast suspension and CFS or YM broth (negative control) were combined as described previously ^1^. G. vaginalis and P. bivia were incubated anaerobically at 37°C in modified BHI broth supplemented with 5% (v/v) horse blood, whereas C. albicans was incubated aerobically at 30°C in YM broth. Samples were collected at 0, 4, 8, 12, 24, and 48 h, serially diluted, and plated for viable cell enumeration.

**Inhibition of Biofilm Formation**

G. vaginalis ATCC 14018, P. bivia ATCC 29303, and C. albicans ATCC 10231 were used as representative vaginal pathogens. Each strain was cultured under optimal conditions and adjusted to 10⁵–10⁶ CFU/mL for G. vaginalis and P. bivia, and 10⁵ CFU/mL for C. albicans. Aliquots (100 μL) of each suspension were dispensed into 96-well microtiter plates. To evaluate inhibitory activity, 100 μL of CFS was added to treatment wells, while control wells received an equal volume of MRS broth (final volume: 200 μL per well). Plates containing G. vaginalis and P. bivia were incubated anaerobically at 37°C for 48 h in modified BHI (mBHI) supplemented with 5% (v/v) horse blood without agitation. C. albicans plates were incubated aerobically at 30°C for 48 h in YM broth without agitation. Following incubation, planktonic cells were removed, and wells were gently washed twice with PBS. Biofilms were stained with 0.1% (w/v) crystal violet for 15 min at room temperature. After washing to remove excess stain, bound dye was solubilized in 200 μL absolute ethanol for 15 min, and absorbance was measured at 600 nm using a microplate reader. All experiments were performed in biological triplicate.

**Sample collection and processing at necropsy**

At necropsy, the mice were anesthetized and euthanized. Vaginal fluid, vaginal tissue, and fecal samples were collected for analysis. Vaginal fluid was obtained by flushing the vaginal lumen 10 times with 50 μL of PBS (GenDPOT, Hanam, Korea) using a pipette, and the fluid was collected in microtubes. Vaginal tissues were bisected; one half was fixed in 10% neutral-buffered formalin (DAEJUNG, Siheung, Korea) for histological examination, and the other half was stored at –70°C (Thermo Scientific, Waltham, MA, USA) for RNA extraction and gene expression analysis. Fecal samples were stored at –70°C and subsequently used for gut microbiota profiling using 16S V3-V4 rRNA gene-based next-generation sequencing (NGS). All animal procedures were conducted at the HLB BioStep animal facility (3rd animal housing unit, Korea) under specific pathogen-free (SPF) conditions. The study protocol was reviewed and approved by the Institutional Animal Care and Use Committee of HLB BioStep (IACUC No. BIOSTEP IACUC 23-KE-0509).

**Quantification of G. vaginalis colonization**

To assess the colonization of *G. vaginalis* in the vaginal tract, vaginal fluid samples were subjected to 10-fold serial dilutions in phosphate-buffered saline (PBS; GenDPOT, Hanam, Korea). Diluted samples were plated onto NYCIII agar and incubated under anaerobic conditions at 37°C for 48 h in an anaerobic jar (Kisanbio, Seoul, Korea). Following incubation, colony-forming units (CFUs) were enumerated on plates containing 30–300 colonies, and the bacterial concentration was expressed as CFU per milliliter.

**Quantification of pro-inflammatory cytokines in vaginal tissue lysates**

Total RNA was extracted from the vaginal tissues using TRIzol reagent (Thermo Scientific, Waltham, MA, USA) in pre-filled bead tubes and homogenized using Bead Ruptor Elite (Omni International, Kennesaw, GA, USA). RNA was isolated using chloroform and isopropanol, followed by ethanol washing and centrifugation at 13,000 rpm for 5 min at 4 °C. After discarding the supernatant, the RNA pellet was dissolved in DEPC water. RNA concentration and purity were measured using a NanoDrop 2000 spectrophotometer (Thermo Scientific, Waltham, MA, USA), and equal amounts of RNA were reverse-transcribed into complementary DNA (cDNA).

cDNA synthesis was performed using ReverTraAce qPCR RT Master Mix with gDNA Remover (Toyobo, Osaka, Japan) according to the manufacturer’s instructions. The thermal cycling protocol was as follows: 37°C for 15 min, 50°C for 5 min, 98°C for 5 min, followed by a hold at 4°C. Quantitative real-time PCR was performed using the CFX Connect Real-Time PCR Detection System (Bio-Rad, Hercules, CA, USA) to measure the expression levels of IL-1β, TNF-α, and GAPDH.

The primer sequences were as follows: IL-1β forward; 5′-TGTCTTGGCCGAGGACTAAGG-3′ and reverse 5′-TGGGCTGGACTGTTTCTAATGC-3′; TNF-α forward 5′-AGAAACACAAGATGCTGGGACAGT-3′ and reverse 5′-CCTTTGCAGAACTCAGGAATGG-3′; and GAPDH forward 5′-AAATGGTGAAGGTCGGTGTGAAC-3′ and reverse 5′-CAACAATCTCCACTTTGCCACTG-3′.

PCR amplification was conducted using qPCRBIO SyGreen Blue Mix Separate-Rox (PCR Biosystems, London, UK) according to the manufacturer’s instructions. The thermal cycling conditions were as follows: initial denaturation at 95°C for 2 min, followed by 40 cycles of 95°C for 5 s, 60°C for 30 s, and 65°C for 5 s, with a final denaturation step at 95°C for 5 s. Relative gene expression was calculated using the $2^{-\Delta{\Delta C}_{T}}$method with GAPDH as the reference gene.

**Microbiota Profiling via 16S rRNA Gene Sequencing**

Genomic DNA was extracted from vaginal fluid and fecal samples using the Mag-Bind Universal Pathogen Kit (Omega Bio-tek, Norcross, GA, USA), following the manufacturer’s protocol. Briefly, fecal pellets and vaginal fluid were suspended in 275 μL of SLX-Mlus Buffer and subjected to mechanical lysis using a MixerMill MM400 bead beater (Retsch, Haan, Germany). DNA was then isolated, purified, and eluted.

The bacterial 16S rRNA gene were amplified using a forward primer (5′-TCGTCGGCAGCGTCAGATGTGTATAAGAGACAGCCTACGGGNGGCWGCAG-3′) and a reverse primer (5′-GTCTCGTGGGCTCGGAGATGTGTATAAGAGACAGGACTACHVGGGTATCTAATCC-3′).

PCR amplification was performed using 2× KAPA HiFi HotStart ReadyMix (Roche, Basel, Basel-Stadt, Switzerland), with an initial denaturation at 95°C for 3 minutes, followed by 25 cycles of 95°C for 30 seconds, 55°C for 30 seconds, and 72°C for 30 seconds, and a final extension at 72°C for 5 minutes.

PCR products were cleaned using HiAccuBeads (AccuGene, Incheon, South Korea) and a magnetic stand. Indexing PCR was then performed using IDT indexing primers (Integrated DNA technologies, Coralville, Iowa, US), 2× KAPA HiFi HotStart ReadyMix, and PCR-grade water. Cycling conditions were: 95°C for 3 minutes, followed by 8 cycles of 95°C for 30 seconds, 55°C for 30 seconds, and 72°C for 30 seconds, with a final extension at 72°C for 5 minutes and a hold at 4°C. After the clean-up step, the concentration of libraries was measured using a Qubit 4.0 fluorometer (Thermo Fisher Scientific, Waltham, MA, USA) with the 1× dsDNA High Sensitivity Assay Kit (Thermo Fisher Scientific, Waltham, MA, USA).

Raw paired-end 16S rRNA gene sequencing reads generated on the MiSeq platform (Illumina, San Diego, CA, USA) were initially demultiplexed based on unique sample-specific barcodes.

Downstream bioinformatics processing was performed using **QIIME2 (v 2021.2)** following standard workflows. Primer trimming, quality filtering, denoising, paired-end read merging, and chimera removal were conducted using the **DADA2 plugin (qiime dada2 denoise-paired)**, to infer **amplicon sequence variants (ASVs)**. Specifically, forward and reverse reads were trimmed at 17 bp and 21 bp, respectively, to remove primer sequences. Based on per-base quality score profiles, reads were truncated at 270 bp (forward) and 220 bp (reverse) to retain high-quality sequences while minimizing error rates. All other DADA2 parameters were maintained at their default settings. Taxonomic classification of ASVs was performed using a **naïve Bayes classifier** trained on the **SILVA reference database (version 138)**. Taxonomic assignments were generated using the **qiime feature-classifier classify-sklearn** plugin with default confidence thresholds. After DADA2 processing, samples with fewer than 15,000 reads were excluded from downstream analyses. As a result, one vaginal fluid sample in the GV+vLH group did not meet this sequencing depth criterion and was excluded, resulting in a final sample size of *n* = 7 for this group, while all other groups contained *n* = 8 samples. Alpha-diversity metrics (Observed, Shannon, and Simpson) were calculated using R packages phyloseq (v 1.46.0) on non-rarefied count data. For beta-diversity feature tables were rarefied to a minimum library size, and community dissimilarity was assessed using multiple distance metrics (Weighted UniFrac, Unweighted UniFrac, Bray-Curtis, and Jaccard) and visualized by principal coordinate analysis (PCoA). Differential abundance analyses were conducted using DESeq2 on non-rarefied count data, as described in the Statistical Analysis section.

Taxonomic assignments were considered reliable when supported by consistent classification confidence across samples. Given the limitations of 16S rRNA gene sequencing, genus-level assignments with low confidence or inconsistent prevalence were excluded from primary analyses, and higher taxonomic ranks were emphasized.

**16S rDNA sequencing of L. helveticus 20838**

Pure cultures of *L. helveticus* 20838 was grown on MRS agar at 37°C for 24 hours. The plate was sent to Macrogen Inc. (Seoul, South Korea) for bi-directional 16S rDNA 2sequencing.

The assembled 16S rRNA gene sequence of Lactobacillus helveticus 20838 was compared against reference sequences using **BLASTn** (Basic Local Alignment Search Tool for nucleotides) ^3^. Sequence similarity searches were performed against the **NCBI 16S ribosomal RNA sequences (Bacteria and Archaea) database**, using default BLASTn parameters. Taxonomic assignment was based on highest sequence identity and alignment coverage. Bi-directional sequencing results were assembled using Codon Code Aligner (Codon Code Corporation, USA) and compared with reference sequences from the GenBank database (PX488011; <https://www.ncbi.nlm.nih.gov/genbank/>).

**Biogenic amine production assay**

LABs were grown on the optimal growth, was streaked out onto special medium with precursor of histamine, cadaverine, tyramine and putrescine according to Bover-Cid and Holzapfel ^4^ and incubated for 4 days at 37°C. After incubation for 4 days, check the change in the color of the medium to determine the positive and negative. *E. coli* ATCC 25922 was used as a positive control.

**Gelatine hydrolysis test**

The basic protocol was followed according to the ASM Science Recommendation ^5^. LABs grown under optimal growth conditions, was inoculated with an inoculation loop into a gelatine medium and incubated at 30°C for up to 5 days and checked daily for gelatine liquefaction and bacterial growth. Gelatine normally liquefies at 28°C and above. To confirm that liquefaction was due to gelatinase activity, the tubes were immersed in a refrigerator for 30 minutes. Afterwards, tubes are tilted to observe if gelatine has been hydrolyzed. Hydrolysis of gelatine will result in a liquified medium even after exposure to cold temperature. *Bacillus cereus* ATCC 11778 was used as a positive control.

**Antibiotic resistance test**

The basic protocol was based on ISO recommendation (ISO-10932, 2010). The broth dilution method was used to assess the minimal inhibitory concentration (MIC) of the strain against antibiotics. In the broth microdilution, test organisms were purely cultivated in broth media, and then all organisms were washed with 1X PBS. The bacterial solution washed in PBS was adjusted to 0.1 – 0.2 of 600nm optical density (OD) units. 10 μl (1 - 3 x 107 CFU/ml) of the strain was inoculated in 96 well plates containing 19 μl of LSM broth media with antibiotics (approximately 1 - 3 x 104 CFU/well). The strain was considered susceptible when it was inhibited at a concentration for a specific antibiotic equal or lower than the established cut-off value and resistant when it was not inhibited at a concentration of a specific antibiotic higher than the established cut-off 4value according to the parameters established by the European Food Safety Authority (EFSA, 2018).

**Possible toxigenic gene detection**

The search for virulence factors in *L. helveticus* 20838 was completed using the VirulenceFinder2.0 Server, which is a component of the publicly available web-based tool for whole-genome sequencing (WGS) analysis hosted by the Center for Genomic Epidemiology (CGE) (http://www.genomicepidemiology.org/). The database system is designed to detect homologous sequences for the virulence genes associated with *E. coli, Enterococcus, Listeria,* and *Staphylococcus aureus* in WGS data ^6^. The output consists of best-matching genes from BLAST analysis of the selected database against the submitted genome of *L. helveticus* 20838. The selected % ID threshold was set at 90.00%, and the selected minimum length at 60.00%. If there is a matching result, the output shows information on the predicted virulence gene, the % ID, the length of query and database gene, the position of the hit in the contig, and the accession number of the hit ^7^.

**Antibiotic resistant gene detection**

After performing whole genome sequencing, we used the “ResFinder” web software to search for antibiotic resistant genes in *L. helveticus* 20838. This web software was recommended by EFSA to find the antibiotic-resistant genes (EFSA, 2018). ResFinder identifies acquired antimicrobial resistance genes and/or chromosomal mutations in total or partial sequenced isolates of bacteria. The selected %ID threshold was set at 90.00%, and the selected minimum length at 60.00%. ResFinder consists of two programs, ResFinder.py identifying acquired genes, and PointFinder.py identifying chromosomal mutations. Software and databases are available online ^8^.

**Whole genome sequencing (WGS) of L. helveticus 20838**

Traditionally, DNA-DNA hybridization (DDH) has served as the gold standard for bacterial species delineation since the 1960s. However, with the advent of high-throughput sequencing technologies, Average Nucleotide Identity (ANI) has emerged as a more robust and widely accepted metric for assessing genomic similarity between prokaryotic strains. The DNA of *L. helveticus* 20838 was extracted after full growth using MagListo Genomic DNA Extraction Kit (Qiagen) following the instructions of the manufacturer. SMRTbell DNA template libraries of 20-kb average insert size for the bacterial samples were prepared according to the manufacturer’s specification, with G-tubes (Covaris) used for fragmentation. SMRT sequencing was carried out on the PacBio RS II according to standard protocols, with the XL binding kit used in conjunction with the C4 sequencing kit. All runs were carried out with diffusion-based loading and analysed using the standard primary data analysis. Raw PacBio long-read sequencing data were processed using the Hierarchical Genome Assembly Process (HGAP) pipeline for de novo assembly, followed by consensus polishing with Quiver. Genome annotation was performed using standard prokaryotic annotation workflows, and assembled sequences were used for downstream comparative genomic and safety analyses. Assembly quality metrics, genome statistics, and annotation results are summarized in Supplementary Tables S1–S3. Source codes for an implementation of HGAP and Quiver, data sets and additional documentation are available at http://www.pacbiodevnet.com/HGAP and <http://www.pacbiodevnet.com/quiver>.

Putative virulence genes were screened using **VirulenceFinder** implemented via the **Center for Genomic Epidemiology (CGE)** web platform. The assembled whole-genome sequence of L. helveticus 20838 was queried against the VirulenceFinder database, which contains curated virulence-associated genes from clinically relevant bacterial pathogens. Searches were performed using default parameters, with a minimum sequence identity threshold of 90% and a minimum coverage threshold of 60%.

Acquired antimicrobial resistance genes were screened using **ResFinder (version 4.1)** implemented via the **Center for Genomic Epidemiology (CGE)** web platform (<https://www.genomicepidemiology.org/>). The assembled whole-genome sequence of Lactobacillus helveticus 20838 was queried against the ResFinder database using default parameters, with a minimum sequence identity threshold of **90%** and a minimum coverage threshold of **60%**. Analyses were performed on **30 April 2025**.

**Table S1.** Comparison of ANI values of *L. helveticus* 20838(A) and *L. helveticus* DPC4571

| **Metric** | **Value (%)** |
| --- | --- |
| ANI comparative value of A and B (%) | 97.87 |
| Genome A length (bp) | 2,193,000 |
| Genome B length (bp) | 2,080,800 |

**Table S2**. Whole genome sequence results of *L. helveticus* 20838 compared to *L.*

*helveticus* DPC4571

| **Contents** | ***L. helveticus* 20838** | ***L. helveticus* DPC4571** |
| --- | --- | --- |
| Status | COMPLETE | COMPLETE |
| No. of contigs | 3 | 1 |
| Plasmids | 1 | 0 |
| Genome size (bp) | 2,194,774 | 2,080,931 |
| DNA G+C content (%) | 36.8 | 37.1 |
| No. of CDSs | 2291 | 2247 |
| No. of RNAs | 75 | 73 |
| Homology with  *L. helveticus* 20838  by OrthoANI analysis (%) |  | 97.87 |

**Table S3.** Whole genome sequence overview of *L. helveticus* 20838

| **Genome** | *Lactobacillus helveticus* 20838 |
| --- | --- |
| **Taxonomy ID** | 1587 |
| **Domain** | Bacteria |
| **Taxonomy** | Bacteria; Bacillati; Bacillota; Bacilli; Lactobacillales; *Lactobacillaceae;*  *Lactobacillus; Lactobacillus helveticus; Lactobacillus helveticus* 20838 |
| **Closest neighbor** | *Lactobacillus helveticus* DPC 4571 |
| **Size (bp)** | 2,194,774 |
| **GC Content in the**  **DNA** | 36.8 mol% G+C |
| **Number of Contigs** | 3 |
| **Number of Coding**  **Sequences** | 2291 |
| **Number of RNAs** | 75 |

**Table S4.** Growth conditions of LABs in ISO10932

| **Species** | **Media** | **Temperature (°C)** | **Atmosphere** | **Incubation time (h)** |
| --- | --- | --- | --- | --- |
| *Bifidobacterium spp.* | MRS-Cystein | 37 | Anaerobic | 24 to 48 |
| *Lactobacillus plantarum* | MRS | 28 | Anaerobic or ambient | 16 to 24 |
| Other *lactobacilli* | MRS | 37 | Anaerobic or ambient | 16 to 24 |

**Table S5.** The composition of biogenic amine detection media for assay

| **Component** | **gram/Liter** |
| --- | --- |
| Tryptone | 5 |
| Yeast extract | 5 |
| Meat extract | 5 |
| Sodium chloride | 2.5 |
| Glucose | 0.5 |
| Tween 80 | 1 |
| MgSO_4_ | 0.2 |
| MnSO_4_ | 0.05 |
| FeSO_4_ | 0.04 |
| Ammonium citrate | 2 |
| Thiamine | 0.01 |
| K_2_PO_4_ | 2 |
| CaCO_3_ | 0.1 |
| Pyridoxal-5-phosphate | 0.05 |
| Amino acid (Ornithine, Lysine, Tyrosine, Histidine) | 10 |
| Bromo cresol purple | 0.06 |
| Agar | 20 |
| pH | 5.3 |

**Table S6.** The partial sequence of *L. helveticus* 20838 16S ribosomal RNA

| **Strain** | **Sequences** |
| --- | --- |
| *L. helveticus* 20838 | GTGCCTAATACATGCAAGTCGAGCGAGCAGAACCAGCAGATTTACTTCGGTAATGACGCTGGGGACGCGAGCGGCGGATGGGTGAGTAACACGTGGGGAACCTGCCCCATAGTCTAGGATACCACTTGGAAACAGGTGCTAATACCGGATAATAAAGCAGATCGCATGATCAGCTTATAAAAGGCGGCGTAAGCTGTCGCTATGGGATGGCCCCGCGGTGCATTAGCTAGTTGGTAAGGTAACGGCTTACCAAGGCAATGATGCATAGCCGAGTTGAGAGACTGAACGGCCACATTGGGACTGAGACACGGCCCAAACTCCTACGGGAGGCAGCAGTAGGGAATCTTCCACAATGGACGCAAGTCTGATGGAGCAACGCCGCGTGAGTGAAGAAGGTTTTCGGATCGTAAAGCTCTGTTGTTGGTGAAGAAGGATAGAGGTAGTAACTGGCCTTTATTTGACGGTAATCAACCAGAAAGTCACGGCTAACTACGTGCCAGCAGCCGCGGTAATACGTAGGTGGCAAGCGTTGTCCGGATTTATTGGGCGTAAAGCGAGCGCAGGCGGAAGAATAAGTCTGATGTGAAAGCCCTCGGCTTAACCGAGGAATTGCATCGGAAACTGTTTTTCTTGAGTGCAGAAGAGGAGAGTGGAACTCCATGTGTAGCGGTGGAATGCGTAGATATATGGAAGAACACCAGTGGCGAAGGCGGCTCTCTGGTCTGCAACTGACGCTGAGGCTCGAAAGCATGGGTAGCGAACAGGATTAGATACCCTGGTAGTCCATGCCGTAAACGATGAGTGCTAAGTGTTGGGAGGTTTCCGCCTCTCAGTGCTGCAGCTAACGCATTAAGCACTCCGCCTGGGGAGTACGACCGCAAGGTTGAAACTCAAAGGAATTGACGGGGGCCCGCACAAGCGGTGGAGCATGTGGTTTAATTCGAAGCAACGCGAAGAACCTTACCAGGTCTTGACATCTAGTGCCATCCTAAGAGATTAGGAGTTCCCTTCGGGGACGCTAAGACAGGTGGTGCATGGCTGTCGTCAGCTCGTGTCGTGAGATGTTGGGTTAAGTCCCGCAACGAGCGCAACCCTTATTATTAGTTGCCAGCATTAAGTTGGGCACTCTAATGAGACTGCCGGTGACAAACCGGAGGAAGGTGGGGATGACGTCAAGTCATCATGCCCCTTATGACCTGGGCTACACACGTGCTACAATGGGCAGTACAACGAGAAGCGAGCCTGCGAAGGCAAGCGAATCTCTGAAAGCTGTTCTCAGTTCGGACTGCAGTCTGCAACTCGACTGCACGAAGCTGGAATCGCTAGTAATCGCGGATCAGAACGCCGCGGTGAATACGTTCCCGGGCCTTGTACACACCGCCCGTCACACCATGGAAGTCTGCAATGCCCAAAGCCGGTGGCCTAACCTTCGGGAAGGAGCCGTCTAAGGCAGGGCAGATGACTGGGGTGAAGTCGTA |

**Table S7.** The 16S rDNA sequence NCBI blast matching result of *L. helveticus* 20838*.*

| **Sample name** | **Description** | **Per. Identity** |
| --- | --- | --- |
| *L. helveticus* 20838 | *Lactobacillus helveticus* DSM20075 = CGMCC 1.1877 16S ribosomal RNA. partial sequence | 99.39% |

**Table S8.** Haemolysis activity of *L. helveticus* 20838 and *B. cereus* ATCC 27348 using 5% sheep blood agar.

| **Strain** | **Haemolysis activity** |
| --- | --- |
| *L. helveticus* 20838 | Gamma (γ) |
| *B. cereus* ATCC 27348  (positive control) | Beta (β) |

**Table S9.** Biogenic amines production activity of *L. helveticus* 20838 and *E. coli* ATCC 25922

| **Test Strains** | **Histamine** | **Cadaverine** | **Tyramine** | **Putrescine** |
| --- | --- | --- | --- | --- |
| *L. helveticus* 20838 | Negative | Negative | Negative | Negative |
| *E. coli* ATCC 25922  (positive control) | Positive | Positive | Positive | Positive |

**Table S10.** Gelatine hydrolysis test for *L. helveticus* 20838 and *B. cereus* ATCC 11778

| **Test Strains** | **Gelatine hydrolysis test** |
| --- | --- |
| *L. helveticus* 20838 | Negative |
| *B. cereus* ATCC 11778  (positive control) | Positive |

**Table S11.** Antibiotic resistance gene detection results in the Resfinder for *L. helveticus* 20838.

| **Antimicrobial** | **Class** | **WGS-predicted phenotype** |
| --- | --- | --- |
| gentamicin | aminoglycoside | No resistance |
| tobramycin | aminoglycoside | No resistance |
| streptomycin | aminoglycoside | No resistance |
| amikacin | aminoglycoside | No resistance |
| isepamicin | aminoglycoside | No resistance |
| dibekacin | aminoglycoside | No resistance |
| kanamycin | aminoglycoside | No resistance |
| neomycin | aminoglycoside | No resistance |
| lividomycin | aminoglycoside | No resistance |
| paromomycin | aminoglycoside | No resistance |
| ribostamycin | aminoglycoside | No resistance |
| unknown aminoglycoside | aminoglycoside | No resistance |
| butiromycin | aminoglycoside | No resistance |
| butirosin | aminoglycoside | No resistance |
| hygromycin | aminoglycoside | No resistance |
| netilmicin | aminoglycoside | No resistance |
| apramycin | aminoglycoside | No resistance |
| sisomicin | aminoglycoside | No resistance |
| arbekacin | aminoglycoside | No resistance |
| kasugamycin | aminoglycoside | No resistance |
| astromicin | aminoglycoside | No resistance |
| fortimicin | aminoglycoside | No resistance |
| spectinomycin | aminocyclitol | No resistance |
| fluoroquinolone | quinolone | No resistance |
| ciprofloxacin | quinolone | No resistance |
| unknown quinolone | quinolone | No resistance |
| nalidixic acid | quinolone | No resistance |
| amoxicillin | beta-lactam | No resistance |
| amoxicillin+clavulanic acid | beta-lactam | No resistance |
| ampicillin | beta-lactam | No resistance |
| ampicillin+clavulanic acid | beta-lactam | No resistance |
| cefepime | beta-lactam | No resistance |
| cefixime | beta-lactam | No resistance |
| cefotaxime | beta-lactam | No resistance |
| cefoxitin | beta-lactam | No resistance |
| ceftazidime | beta-lactam | No resistance |
| ertapenem | beta-lactam | No resistance |
| imipenem | beta-lactam | No resistance |
| meropenem | beta-lactam | No resistance |
| piperacillin | beta-lactam | No resistance |
| piperacillin+tazobactam | beta-lactam | No resistance |
| unknown beta-lactam | beta-lactam | No resistance |
| aztreonam | beta-lactam | No resistance |
| cefotaxime+clavulanic acid | beta-lactam | No resistance |
| temocillin | beta-lactam | No resistance |
| ticarcillin | beta-lactam | No resistance |
| ceftazidime+avibactam | beta-lactam | No resistance |
| penicillin | beta-lactam | No resistance |
| ceftriaxone | beta-lactam | No resistance |
| ticarcillin+clavulanic acid | beta-lactam | No resistance |
| cephalothin | beta-lactam | No resistance |
| piperacillin+clavulanic acid | beta-lactam | No resistance |
| ceftiofur | under_development | No resistance |
| sulfamethoxazole | folate pathway antagonist | No resistance |
| trimethoprim | folate pathway antagonist | No resistance |
| fosfomycin | fosfomycin | No resistance |
| vancomycin | glycopeptide | No resistance |
| teicoplanin | glycopeptide | No resistance |
| bleomycin | glycopeptide | No resistance |
| lincomycin | lincosamide | No resistance |
| clindamycin | lincosamide | No resistance |
| dalfopristin | streptogramin a | No resistance |
| pristinamycin iia | streptogramin a | No resistance |
| virginiamycin m | streptogramin a | No resistance |
| quinupristin+dalfopristin | streptogramin a | No resistance |
| tiamulin | pleuromutilin | No resistance |
| carbomycin | macrolide | No resistance |
| erythromycin | macrolide | No resistance |
| azithromycin | macrolide | No resistance |
| oleandomycin | macrolide | No resistance |
| spiramycin | macrolide | No resistance |
| tylosin | macrolide | No resistance |
| telithromycin | macrolide | No resistance |
| tetracycline | tetracycline | No resistance |
| doxycycline | tetracycline | No resistance |
| minocycline | tetracycline | No resistance |
| tigecycline | tetracycline | No resistance |
| quinupristin | streptogramin b | No resistance |
| pristinamycin ia | streptogramin b | No resistance |
| virginiamycin s | streptogramin b | No resistance |
| linezolid | oxazolidinone | No resistance |
| chloramphenicol | amphenicol | No resistance |
| florfenicol | amphenicol | No resistance |
| colistin | polymyxin | No resistance |
| fusidic acid | steroid antibacterial | No resistance |
| mupirocin | pseudomonic acid | No resistance |
| rifampicin | rifamycin | No resistance |
| metronidazole | nitroimidazole | No resistance |
| narasin | ionophores | No resistance |
| salinomycin | ionophores | No resistance |
| maduramicin | ionophores | No resistance |

**Table S12.** Possible virulence gene detection results of *L. helveticus* 20838 in CGE virulence finder.

|  | Virulence genes for *Enterococcus* | Virulence genes for *Listeria* | Virulence genes for *Escherichia coli* | Shiga-toxin genes | Exoenzyme genes for *S. aureus* | Hostimm genes for *S. aureus* | Toxin genes for *S. aureus* |
| --- | --- | --- | --- | --- | --- | --- | --- |
| Virulence factor | Not hit found | Not hit found | Not hit found | Not hit found | Not hit found | Not hit found | Not hit found |
| Identity |  |  |  |  |  |  |  |
| Query/  template length |  |  |  |  |  |  |  |
| Contig |  |  |  |  |  |  |  |
| Position in contig |  |  |  |  |  |  |  |
| Protein function |  |  |  |  |  |  |  |
| Accession number |  |  |  |  |  |  |  |

References

1. Wang, S., Wang, Q., Yang, E., Yan, L., Li, T., and Zhuang, H. (2017). Antimicrobial Compounds Produced by Vaginal Lactobacillus crispatus Are Able to Strongly Inhibit Candida albicans Growth, Hyphal Formation and Regulate Virulence-related Gene Expressions. Front Microbiol *8*, 564. 10.3389/fmicb.2017.00564.

2. Shang, X., Bai, H., Fan, L., Zhang, X., Zhao, X., and Liu, Z. (2024). In vitro biofilm formation of Gardnerella vaginalis and Escherichia coli associated with bacterial vaginosis and aerobic vaginitis. Front Cell Infect Microbiol *14*, 1387414. 10.3389/fcimb.2024.1387414.

3. Altschul, S.F., Gish, W., Miller, W., Myers, E.W., and Lipman, D.J. (1990). Basic local alignment search tool. J Mol Biol *215*, 403-410. 10.1016/s0022-2836(05)80360-2.

4. Bover-Cid, S., and Holzapfel, W.H. (1999). Improved screening procedure for biogenic amine production by lactic acid bacteria. Int J Food Microbiol *53*, 33-41. 10.1016/s0168-1605(99)00152-x.

5. Echave, M.C., Saenz del Burgo, L., Pedraz, J.L., and Orive, G. (2017). Gelatin as Biomaterial for Tissue Engineering. Curr Pharm Des *23*, 3567-3584. 10.2174/0929867324666170511123101.

6. Joensen, K.G., Scheutz, F., Lund, O., Hasman, H., Kaas, R.S., Nielsen, E.M., and Aarestrup, F.M. (2014). Real-time whole-genome sequencing for routine typing, surveillance, and outbreak detection of verotoxigenic Escherichia coli. J Clin Microbiol *52*, 1501-1510. 10.1128/jcm.03617-13.

7. Kim, M.J., Ku, S., Kim, S.Y., Lee, H.H., Jin, H., Kang, S., Li, R., Johnston, T.V., Park, M.S., and Ji, G.E. (2018). Safety Evaluations of Bifidobacterium bifidum BGN4 and Bifidobacterium longum BORI. Int J Mol Sci *19*. 10.3390/ijms19051422.

8. Clausen, P., Aarestrup, F.M., and Lund, O. (2018). Rapid and precise alignment of raw reads against redundant databases with KMA. BMC Bioinformatics *19*, 307. 10.1186/s12859-018-2336-6.
